# Large-scale atomistic and quantum-mechanical simulations of a Nafion membrane: Morphology, proton solvation and charge transport

**DOI:** 10.3762/bjnano.4.65

**Published:** 2013-09-26

**Authors:** Pavel V Komarov, Pavel G Khalatur, Alexei R Khokhlov

**Affiliations:** 1 Institute of Organoelement Compounds, RAS, Moscow 119991, Russia; 2 Department of Theoretical Physics, Tver State University, Tver 170002, Russia; 3 Institute for Advanced Energy Related Nanomaterials, Ulm University, Ulm D-89069, Germany; 4 Physics Department, Moscow State University, Moscow 119991, Russia

**Keywords:** atomistic simulation, morphology, Nafion membrane, proton transport, quantum molecular dynamics

## Abstract

Atomistic and first-principles molecular dynamics simulations are employed to investigate the structure formation in a hydrated Nafion membrane and the solvation and transport of protons in the water channel of the membrane. For the water/Nafion systems containing more than 4 million atoms, it is found that the observed microphase-segregated morphology can be classified as bicontinuous: both majority (hydrophobic) and minority (hydrophilic) subphases are 3D continuous and organized in an irregular ordered pattern, which is largely similar to that known for a bicontinuous double-diamond structure. The characteristic size of the connected hydrophilic channels is about 25–50 Å, depending on the water content. A thermodynamic decomposition of the potential of mean force and the calculated spectral densities of the hindered translational motions of cations reveal that ion association observed with decreasing temperature is largely an entropic effect related to the loss of low-frequency modes. Based on the results from the atomistic simulation of the morphology of Nafion, we developed a realistic model of ion-conducting hydrophilic channel within the Nafion membrane and studied it with quantum molecular dynamics. The extensive 120 ps-long density functional theory (DFT)-based simulations of charge migration in the 1200-atom model of the nanochannel consisting of Nafion chains and water molecules allowed us to observe the bimodality of the van Hove autocorrelation function, which provides the direct evidence of the Grotthuss bond-exchange (hopping) mechanism as a significant contributor to the proton conductivity.

## Introduction

The hydrogen-based power engineering is one of the most important technologies of clean energy. The main problem here is to design efficient fuel cells (FC), electrochemical devices that transform hydrogen fuels into electric power avoiding combustion processes that proceed with large energy loss [1]. The earliest fuel cells based on proton exchange membrane (PEM), consisting of a copolymer of sulfonated polystyrene and divinylbenzene, served as the power plants for the Gemini space missions in the early 1960s. Now, PEM fuel cells show the greatest, most immediate, and most widespread potential applications and are considered as a promising power source for portable devices, home power plants and vehicles.

Typically, PEM is a polymer material, which is a key component of polymer electrolyte fuel cells (PEFCs). The polymer membrane provides a conducting medium for transferring positively charged hydrogen ions (protons) from the anode to the cathode; also, it serves as a barrier to fuel gas cross-leaks and electrical insulation between the electrodes.

On the anode side, hydrogen fuel diffuses to the anode catalyst where it dissociates into electrons *e*^–^ and protons H^+^: H_2_ ↔ 2H^+^ + 2*e*^–^. The hydrated polymer membrane behaves as a solid electrolyte: it swells in the presence of water and passes through into cathode compartment only positively charged protons. On the cathode catalyst, they react exothermically with oxygen molecules and electrons (which have traveled through the external circuit) to form water, 4H^+^ + O_2_ + 4*e*^–^ ↔ 2H_2_O, while electrons travel through the external circuit to produce electric current. Because the overall reaction taking place in a PEFC is 2H_2_ + O_2_ ↔ 2H_2_O, the only waste product is water vapor. The structural organization of PEM determines to a large extent the process of proton transfer from anode to cathode, which is responsible for overall FC efficiency [1].

The membranes are manufactured from special polymers containing both nonpolar atom groups and a relatively small number of polar groups that can dissociate in the water environment to give ions. Such polymers are called ionomers (they are a variety of polyelectrolytes). Up to now, the perfluorosulfonic acid (PFSA) polymers, such as Nafion developed by DuPont in the late 1960s are the most successful PEM materials due to their excellent mechanical properties, chemical stability, and high proton conductivity (5 × 10^−2^ S/cm at 23 °C) [2]. For a long time, Nafion is regarded as a benchmark material in PEFCs due to its excellent combination of conductivity and chemical stability [2–4].

**Figure 1:**
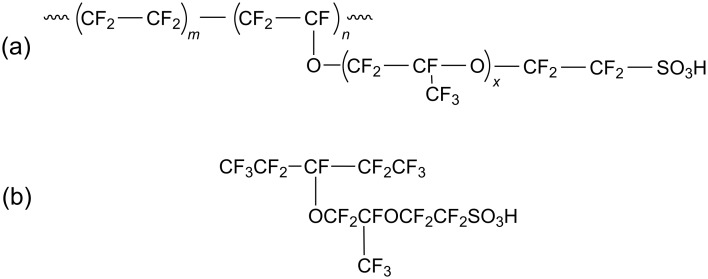
(a) Nafion chain. (b) Nafion sulfonated monomer.

Nafion is a copolymer composed of the fluorocarbon backbone with attached side chains terminated with sulfonic functional groups (Figure 1a).

The backbone of Nafion is similar to that of common Teflon [poly(tetrafluoroethylene)] showing pronounced hydrophobic properties. (Teflon is not only insoluble in water but even not wetting with water.) On the contrary, the polar sulfonic acid groups SO_3_H are strongly hydrophilic and tend to attract water. In the presence of water, they dissociate and deliver protons that serve as charge carriers. Due to the competition of hydrophobic and hydrophilic interactions in a water environment, ionomers tend to separate into a hydrophobic polymer-rich matrix and water-rich nanochannels embedded in the matrix. The details of this process and the organization of the nanochannels are not well understood at the present time. It is assumed that the walls of the hydrophilic channels contain mainly dissociated sulfonic acid groups and their counter ions enabling proton conductivity of the PEM while the hydrophobic domains maintain its mechanical stability [2]. Owing to this specific microphase separated morphology, Nafion and similar nanostructured materials are widely used not only in fuel cell manufacture but also in organic batteries, for water purification by reverse osmosis, etc.

Although the microphase-separated morphology of water-containing PEMs is clearly evidenced by numerous experiments and widely accepted, the detailed information on the resulting nanostructure at molecular level and the mechanism of the proton transport are the subject of active discussions in the current literature (see, for example [5–12]). The importance of optimizing the morphology becomes clear in the following example. In order to achieve high proton conductivity, the molecular structure should be such that the hydrated membrane contains wide interconnected water channels, but these might deteriorate the mechanical characteristics of the membrane. From a general point of view it is clear that the structural organization of the hydrated membrane under different physicochemical conditions is determined by the balance between the elastic deformation of Nafion chains in hydrophobic phase and various intermolecular interactions. Equally important is the understanding of the molecular basis of electrochemical reactions and related to them degradation processes that occur at all stages of the PEFC operation. Many fundamental issues in these fields can be solved using multiscale computer simulations.

Computer simulations are the most consistent theoretical methods as a starting point in understanding such complex systems as PEMs. In principle, Nafion morphologies, which develop as a result of molecular interactions and processing conditions, and other functional properties of PEMs can be modeled on different levels. In particular, a large amount of simulation work has already been carried out over the past decade in an attempt to characterize the morphology of hydrated Nafion membranes at the atomistic scale [13–28]. The total number of atoms in these systems was limited to about two million because of computer memory and CPU limitations [28]. Significant efforts have been made to take into account quantum effects and chemical reactions within the molecular dynamics models. For this purpose, the so-called Reax force field (ReaxFF) [29–30] and empirical valence bond (EVB) methods [31–33] were applied to simulate Nafion. Explicit proton and charge delocalization of the excess proton transport were treated on the basis of the self-consistent multistate empirical valence bond (SC–MS–EVB) method [34–36]. In addition to the atomistic MD simulations mentioned above, there are also mesoscale models in the literature. They include coarse-grained particle-based models widely used in dissipative particle dynamics (DPD) simulations [37–47] and continuum field-theoretic models in which local density fields are employed as collective variables for simulating the structural evolution of phase-separation morphologies [11,48–53]. Several different quantum mechanics approaches have been used in attempts to understand electronic structure and proton conduction in PEFCs [54–59]. There are excellent reviews that cover this subject in considerable detail [5,12,60].

In this paper, the atomistic and first-principles molecular dynamics simulations are employed to investigate the structure formation in hydrated Nafion membrane and the solvation and transport of protons in the water channel of the membrane.

## Results and Discussion

### Atomistic molecular dynamics

#### Model and simulation technique

**Figure 2:**
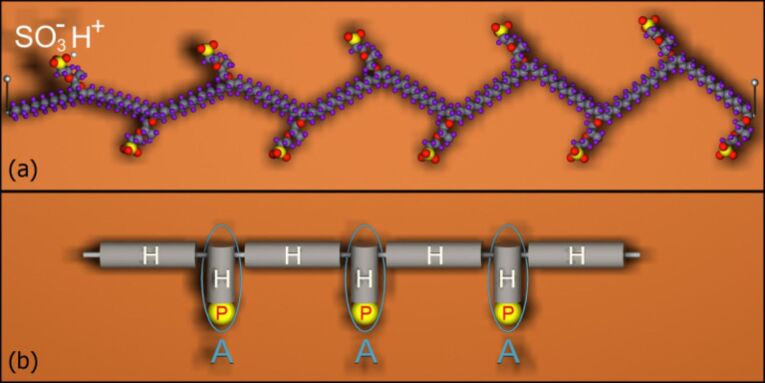
(a) Fragment of a Nafion chain with sulfonic acid groups in dissociated state. The side chains are periodically separated by 15 CF_2_ groups (*m* = 7 in Figure 1a). This corresponds to an average equivalent weight (EW) of Nafion, which is about 1150 g/mol (EW is defined by the number of grams of dry Nafion per mole of sulfonic acid groups, when the material is in the acid form, and is given by the relation EW = 100*m* + 446). A 1100 EW holds for the standard commercialized Nafion 117. All sulfonic acid groups of Nafion are supposed to be in dissociated state. (b) Schematic representation of Nafion as a hydrophobic-hydrophilic copolymer with amphiphilic (A) units. Hydrophilic (P) sites are depicted in yellow and hydrophobic (H) chain sections are shown in gray. Connected H and P groups are considered as an amphiphilic HP "dipole".

As an atomistic model of a hydrated Nafion membrane, we simulated a system consisting of *n*_c_ identical Nafion chains with *n*_s_ = 10 sulfonate groups (Figure 2) and *n*_w_ water molecules. These species were placed in a cubic box with periodic boundary conditions.

All sulfonic acid groups of Nafion were supposed to be in dissociated state (Figure 2a) and hence they had negative charge, while released protons were bound to water molecules to give hydronium ions Н_3_О^+^ responsible for charge transfer reactions.

The hydration level, [H_2_O]/[SO_3_H], is defined as the ratio of the number of water molecules to the number of sulfonated groups: λ = *n*_w_/*n*_s_*n*_c_. The value of λ was 5, 10, and 20. Note that the hydration level λ = 5 is close to the percolation threshold value for water molecules in a Nafion membrane [38,48], while λ = 10 and 20 is close to the operating regime. At these hydration levels, the membrane may function as a proton-conducting material [61–63]. One should keep in mind that the range of λ values corresponding to the specified hydration regime (low or high hydration conditions) depends on the ratio of hydrophobic/hydrophilic segments in a polymer chain [53]. That is why under certain conditions the state with λ = 10 and 20 can be referred to the high hydration level. For the systems under study, both λ values correspond to high acidities (pH<<1). Thus, the chosen λ values correspond to concentrated solutions of strong acids.

For atomistic molecular dynamics (AMD) simulations we used the LAMMPS software package [64] in its highly optimized form for hybrid CPU/GPU nodes so that the computations scaled almost linearly on a massively parallel supercomputer. The force field has a significant effect on the results of any atomistic MD simulation. In this work, a class II (second-generation) polymer-consistent force field (PCFF) [65] was employed. PCFF is an ab-initio based force field in which the total potential energy of an atomistic system is represented as a sum of the following terms: valence terms (the energy contributions for bond, bond angle, torsion, and out-of-plane angle coordinates as well as the energy contributions for diagonal and off-diagonal cross-coupling terms between internal coordinates) and intra- and intermolecular non-bonded interaction terms (a Lennard–Jones "9-6" potential for the van der Waals interactions and a Coulombic term for electrostatic interactions). We used the same force field both for the neutral and the charged species (hydronium cations and sulfonic acid anions SO_3_^−^). For the neutral species, Coulomb interactions in PCFF have been parameterized with charge increments or nonzero atom type charges. For positively/negatively charged ions, charge assignment was done by the charge equilibration method after specifying an overall net charge for the whole structure or the corresponding atomic groups.

Simulation details are identical to those described in our work [27]. Integration step was 1 fs. A 12 Å cut-off radius was applied for Coulomb and van der Waals interactions. The electrostatic interactions were treated by using the PPPM method with a precision of 10^−6^. The dielectric constant was set to 1. All the simulations described in this study were performed in an isobaric-isothermal (NPT) ensemble at *T* = 300 K and *P* = 1 atm, using the Nose–Hoover coupling algorithm with relaxation constants of 0.1 and 0.5 ps for the thermostat and barostat, respectively.

It is well known that the accuracy of any atomistic simulation increases if the size of the material sample is sufficiently large. For hydrated ionomer materials capable of segregating under different conditions, this means that we should operate with the number of atoms about several millions in order to adequately reproduce the system morphology. Another crucial point in any MD study is fully equilibrating the simulated amorphous structure. Therefore our simulations were organized as follows. At the first step during 200 ns, a relatively small system was simulated with the total number of atoms *N* = 65,608 (64 Nafion chains). To avoid metastable structures due to local energy minima trapping, we generated several samples. Each initial configuration was created as a random distribution of Nafion chains, water molecules and hydronium ions. In the initial state we did not build our systems at a density equal to the experimental one. Instead, not to start with strongly entangled chains, our initial configurations were generated randomly at a density far below the experimental reference. After the energy minimization, the final density of these configurations was subsequently refined during a long relaxation under NPT conditions. Next, the simulation box was replicated twice along three directions and the resultant system of *N* = 524,864 atoms (512 Nafion chains) was simulated during 200 ns after a long relaxation. The subsequent replication of the system led to the final system of *N* = 4,198,912 atoms (4096 chains, the initial box size 356^3^ Å^3^). The run of 200 ns was used to obtain the completely equilibrated system, which was also simulated within the time interval of 200 ns. The system density after equilibration ranged from 1.59 to 1.85 g/cm^3^, depending on hydration level. As λ increases, the membrane "swells" more and its density decreases.

The computations reported in this study were performed on the massively parallel supercomputer "Lomonosov" (at Moscow State University) which is based on a hybrid blade system TB2/TB1.1/TB2-TL from T-Platforms equipped with 4,446 X86 compute nodes (Intel Xeon X5570/X5670 2.93 GHz CPUs, 35 568 processor cores), 1554 512-core Tesla X2070 GPUs from Nvidia, and Infiniband QDRinterconnect. Most of our MD simulations were carried out in parallel on 512 hybrid (CPU/GPU) nodes.

#### Morphology

**Figure 3:**
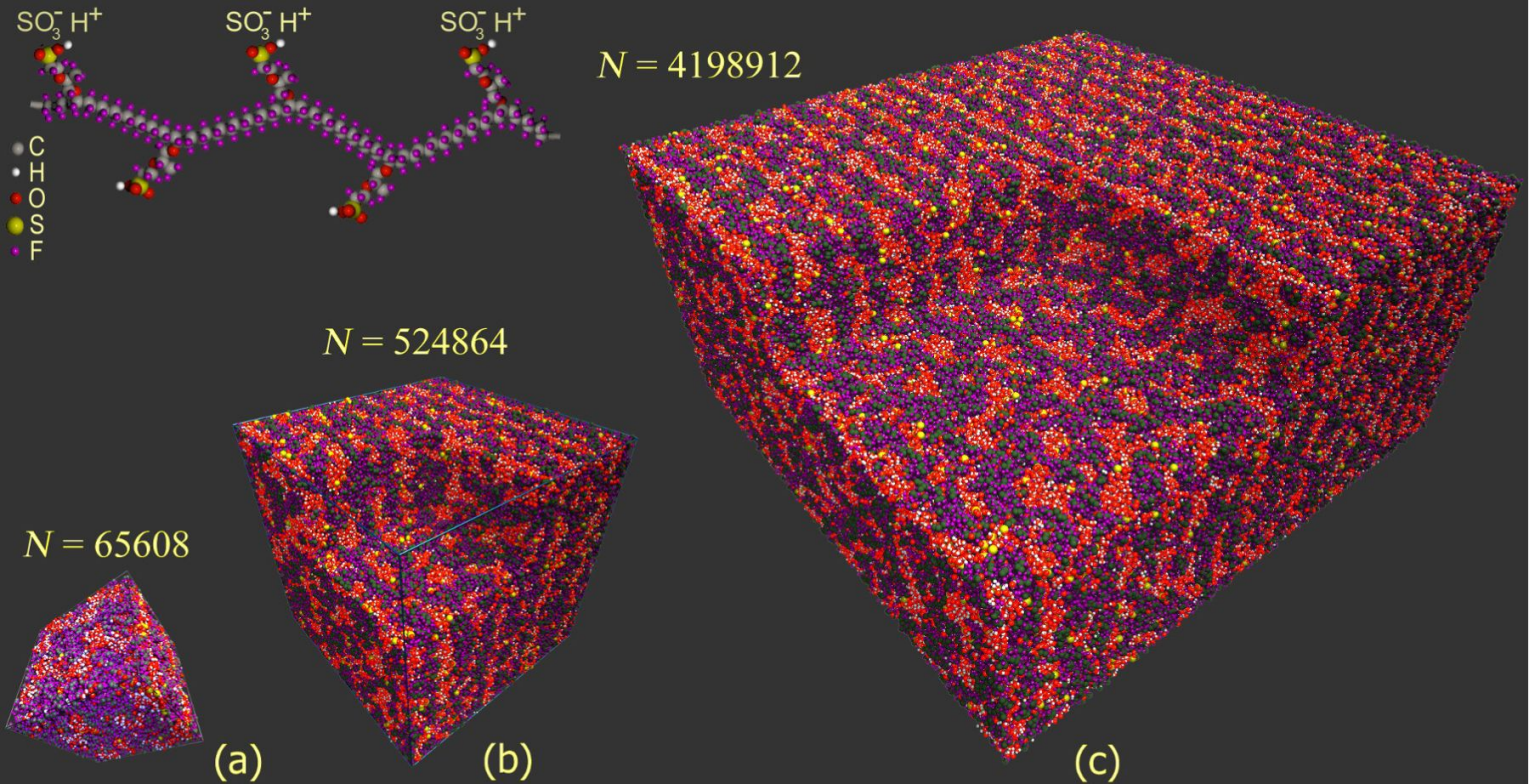
Typical structures predicted by fully atomistic molecular dynamics simulations for hydrated Nafion membrane (10 water molecules per SO_3_^−^ group) and a fragment of Nafion chain. In the systems studied at ambient temperature and under atmospheric pressure (NPT ensemble), all SO_3_H groups are dissociated, while released protons are bound to water molecules to give hydronium ions H_3_O^+^. The total number of atoms *N* in a simulation box with periodic boundary conditions is specified for each system. A portion of materials is removed from the images of systems (b) and (c) for a better understanding of the structural organization. The evolution of each system was monitored for 200 ns after relaxation.

As an example, Figure 3 shows typical snapshots obtained from the atomistic simulation of the three systems studied at λ = 10 and *T* = 298 K.

The MD simulations demonstrated that a hydrated Nafion membrane becomes phase segregated into hydrophobic and hydrophilic domains at all hydration levels investigated. There is a well pronounced network of hydrophilic nanochannels resulting from self-organization and formed by water, hydronium ions, and negatively charged SO_3_^−^ groups. It is seen from Figure 3 that the channels show a tendency to a lamella-like arrangement within several nearest layers (a "curly" lamellar structure). However, the regular spatial arrangement of the channels disappears at the level of far removed layers (at length scales of several nanometers). The hydrophobic matrix permeated by ion-conducting hydrophilic channels is formed mainly by the nonpolar backbones of chains comprising carbon and fluorine atoms. Importantly, the formation of a continuous and percolated aqueous subphase was observed in our simulation even at λ = 5. This observation is confirmed by a number of other theoretical studies of hydrated Nafion. Using the formalism of pair-connectedness correlation functions within the framework of the self-consistent integral equation pRISM theory, we have previously shown that the percolation transition associated with the formation of a continuous water cluster through the entire system occurs near λ ≈ 3 [48]. This is also in keeping with the MD results by Dupuis et al. [22] who found that a λ-value of 5–6 is close to the percolation threshold.

Additional information related to the formation of conductive channels and their topology was obtained from analysis of slices through the hydrophilic subphase (for more detail, see [27]). It was found that the hydrophilic subphase is organized like a sponge-like network and the global membrane morphology can be classified as bicontinuous: both the hydrophilic and the hydrophobic subphase are continuous in space. In other words, both for the hydrophobic-rich regions and hydrophilic-rich regions, one can trace a path from any side of the simulation box to any other side of the box, through one single phase. So both *majority* (hydrophobic) and *minority* (hydrophilic) phases have percolating network-like structures, although the spatial distribution of microdomains does not appear to visually conform to simple periodic shapes. The water network is, of course, not a static entity, but a dynamic system whose shape can change as the water molecules and the local hydrophobic environment diffuse.

**Figure 4:**
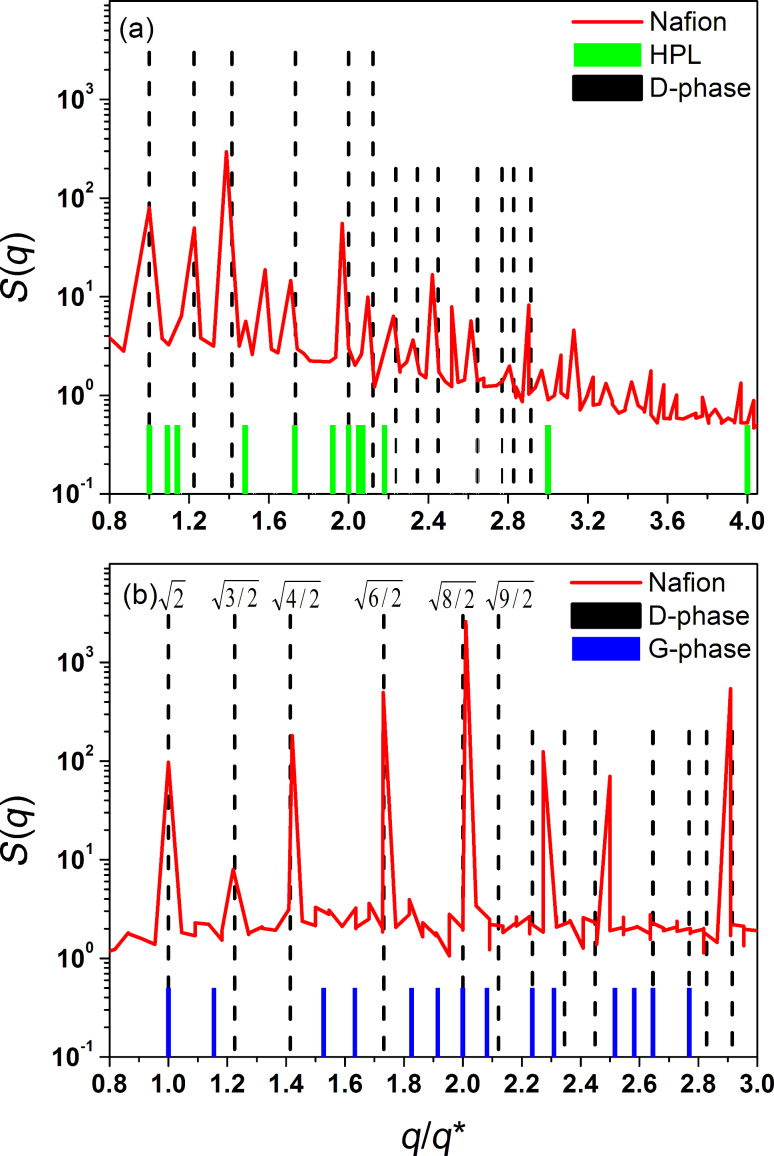
Partial structure factors, *S*(*q*), of the water phase (red line) calculated for (a) the 524,864-atom system and (b) the 4,198,912-atom system at λ = 10 and *T* = 298 K. These functions are compared to those known for a perfect structure, including hexagonally perforated lamellae (HPL), double diamond (DD) and double gyroid (DG). The vertical lines correspond to the expected peaks for these structures [66–69]. The *S*(*q*) functions are plotted versus *q*/q*, where *q* is the wave number and q* is the position of the first, most intensive maximum of the *S*(*q*). The peaks are shown at each magnitude of the scattering vector 
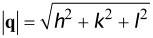
, where *h*, *k*, and *l* are the Miller indices associated with the allowable reflections. (a) The solid (green) and dashed (black) vertical lines correspond to the expected harmonics for *P*6_3_/*mmc* (consistent with the HPL structure) and 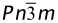 (consistent with DD structure) crystallographic symmetries. (b) The vertical lines correspond to the expected harmonics for 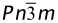 (consistent with DD structure) and 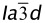 (consistent with DG structure) crystallographic symmetries.

In order to further characterize the predicted microphase-separated structure, we calculated for each simulated system the partial structure factors 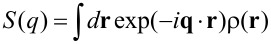 which are the Fourier transform of the partial densities ρ within the simulation box. Of particular interest is the structure factor of the water phase responsible for the formation of ion-conducting channels. These structure factors were compared to those known for different "perfect" morphologies. Several perfect morphologies were considered as possible candidates. First, a simple lamellar (LAM) morphology and hexagonally perforated lamellae (HPL) were considered as candidate phases. The HPL structure consists of alternating minority and majority component layers (that is the hydrophilic and hydrophobic species in our case) in which hexagonally packed domains of majority components extend through the minority component [66]. Possible bicontinuous architectures are naturally associated with well known bicontinuous cubic phases (BCPs). Among many BCPs found in block copolymers and concentrated surfactant systems, the double-diamond (DD) structure with the space group 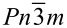, and the Schoen's double gyroid (DG structure with space group 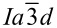) are most common [67–69]. These structures are the most probable candidates and were used for comparison. The results are shown in Figure 4, where we compare the partial structure factors *S*(*q*) calculated for the water phase with those known for HPL, DD and DG morphologies [66–69].

First of all, we would like to draw attention to the fact that the positions of the main harmonics predicted for the three systems with different total number of atoms practically coincide. From inspection of Figure 4, we see that the partial structure factor *S*(*q*) has several distinct peaks. This means that the microphase separation leads to the development of an ordered or partly ordered structure. Next, we analyze the positions of these peaks which correspond to the various harmonics.

One can conclude that the spatial organization of hydrated Nafion is drastically different from that expected for the LAM and HPL mesophases for which the main harmonics are spaced at a ratio of 1:2:3:4… There are also significant differences between the simulated structure and the DG morphology. On the other hand, except for several very weak peaks, most of the intense peaks observed for the 4,198,912-atom system closely resemble the ones expected for 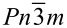 crystallographic symmetry. Indeed, the Bragg reflections of a perfect structure with 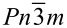 symmetry are characterized by scattering intensities in the relative (scattering vector) positions 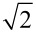, 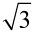, 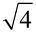, 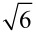, 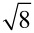, 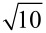, 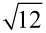, and 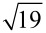, while the eight main harmonics predicted in our simulation are spaced at a *q*/q* ratio of 1.0:1.22:1.42:1.73:2.01:2.25:2.49:2.90 (cf. Figure 4b). As seen, there is a fairly good agreement between the positions of these peaks. However, the peaks at the relative positions 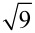, 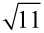, 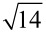, 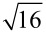, and 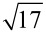 expected for a structure with 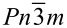 space group are absent or almost invisible. Taking this into account, one has to conclude that microphase-separated Nafion does not form a perfect DD phase, indicating the large amount of various defects present in the simulated structure. These structural defects include a considerable number of undulations and perforations, as can be seen from the examination of numerous snapshots similar to those shown in Figure 3. Nevertheless, the overall organization averaged over many configurations is best described by a pattern, which is consistent with bicontinuous DD morphology. Note that the DD-like mesophases are rather typical for both triblock copolymer melts and amphiphile/water systems [70–71]. In particular, bicontinuous cubic phases (e.g., DD and DG) and transitions between them are often observed for mixtures of water with surfactants or lipids [71].

The observed specific structural organization of hydrated Nafion can be understood by reference to simple models of amphiphilic copolymers [72–75]. Indeed, Nafion is a typical polyamphiphile: its perfluorosulfonic acid macromolecule contains a strongly hydrophobic backbone and less hydrophobic side chains having strongly polar end group. Furthermore, it is known that the association of the hydrated proton with Nafion also suggests its amphiphile-like behavior [35]. From this viewpoint, a hydrophobic-amphiphilic (HA) model of Nafion can schematically be depicted like that shown in Figure 2b. In this model, each amphiphilic unit is a hydrophobic-polar (HP) "dipole" that corresponds to the short side chain of Nafion. The two antagonistic groups, H and P, in the HP "dipole" are repelling each other, but they have to "get along" with each other due to the chemical connectivity, so that the amphiphilic unit prefers to be localized at the H/P boundary, rather than in H- or P-bulk phase. This means that the HP unit possesses a significant surface activity and behaves as an interface modifier (surfactant). This behavior should be seen particularly clearly in the presence of one more polar component, namely water. The tendency to form interfaces between different domains is the key to understand the phase behavior of polyamphiphiles. It has been demonstrated that this feature can lead to fairly specific scenarios of self-organization in the system of amphiphilic-nonpolar copolymers [74–76]. In particular, an amphiphilic component with very incompatible H and P units can form thin channels and slits permeating through a matrix of a majority hydrophobic component [76]. The origin of such morphologies has been discussed for these materials and the physical forces responsible are well recognized. Essentially interfacial tension controls their morphologies. The interfacial tension tends to decrease interfacial stretching, thereby leading to the formation of highly curved monolayers consisting of amphiphilic units with their polar groups in contact with water and hydrophobic groups shielded from contacting the water. When the concentration of these units is not too high, the monolayers are folded in such a way as to form a pattern of connected water channels. Such a behavior is typical for surfactants, lipids, polymeric foams, and assemblies of soap bubbles. In principle, the same percolating structures we observe for our model membrane. In this respect, the structure of hydrated Nafion closely resembles the bicontinuous cubic phase well-known for lyotropic mesophases. The bicontinuous morphology is an especially attractive one for many applications requiring a high interfacial area, such as fuel cell membranes, organic solar cells, etc. In particular, it is clear that such a morphology of the microphase-separated system should be favorable for ionic conductivity.

From the partial structure factor *S*(*q*) we can estimate the characteristic size of water-containing channels and pores in which ion transfer occurs in a Nafion polymeric membrane. The position of the first peak in the structure factor, q*, provides a measure of the average size of water channels, *d* = 2π/q*. For λ = 5, 10 and 20, this measure gives *d* = 25.5, 39.6, and 50.1 Å, respectively, in reasonable agreement with the available experimental data for hydrated Nafion [8]. As seen, our simulation predicts that the effect of the water content on *d* is well pronounced: the micro-size of channels drastically increases with hydration level, the behavior commonly observed in experimental studies.

**Figure 5:**
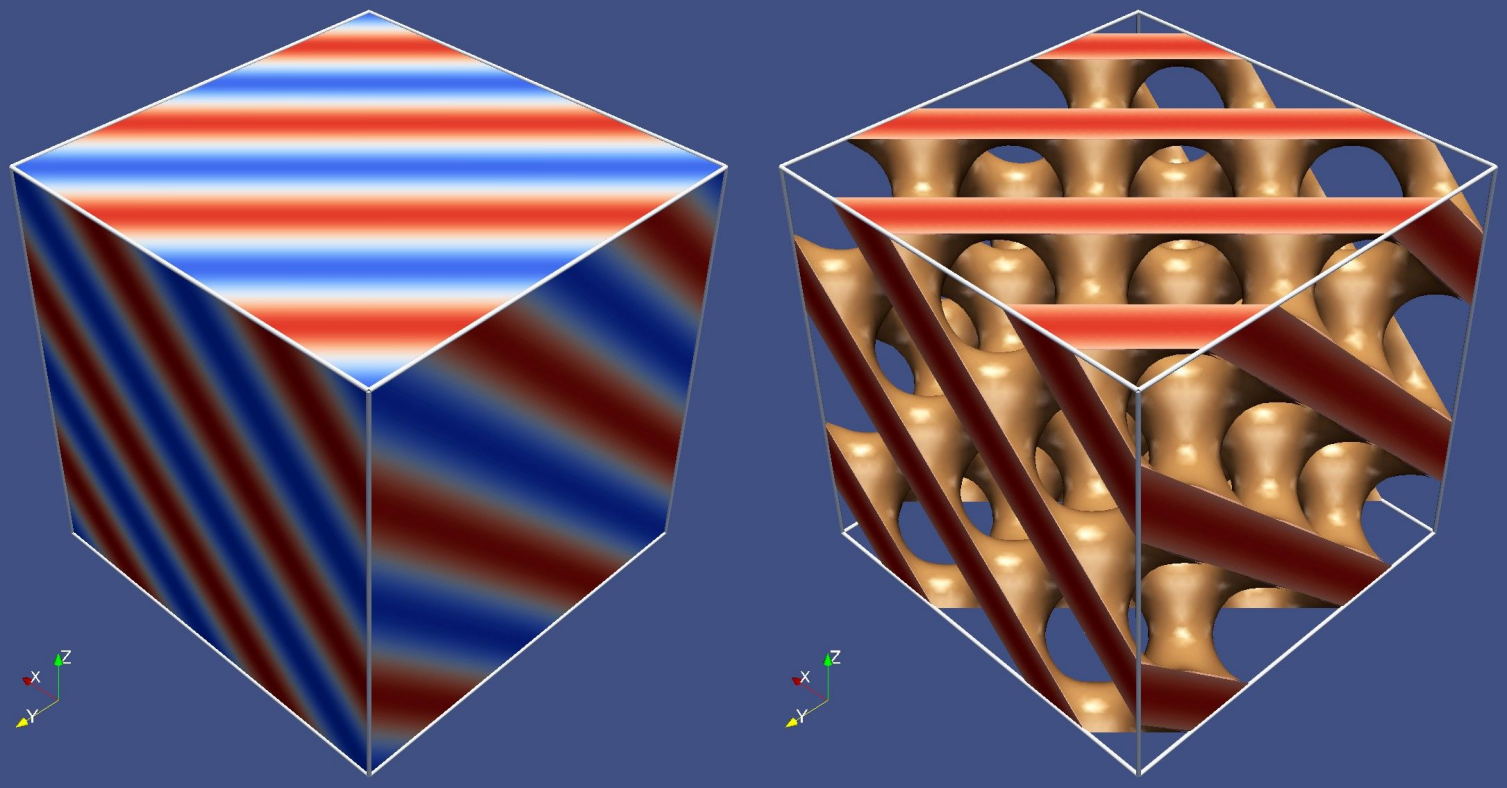
Ordered bicontinuous double diamond structure (space group 224), which contains two separate, connected, triply periodic, tetrahedrally coordinated networks comprised of the minority (A, "red") component in a matrix of the majority (B, "blue") component. Both the red and blue domains are three-dimensionally continuous. The right part of the figure shows the minority component bound by the isodensity surface.

Finally, we should explain why the snapshots of the simulated systems clearly demonstrate a layered structure (Figure 3), while the analysis of structure factors reveals a bicontinuous DD-like morphology (Figure 4). The answer is surprisingly simple. In Figure 5 we present the density distribution in a bicomponent system corresponding to a geometrically perfect DD structure (the OBDD microdomain morphology). It is seen that at any slice, the DD structure looks like a regular layered pattern, in a manner similar to that seen in Figure 3.

**Figure 6:**
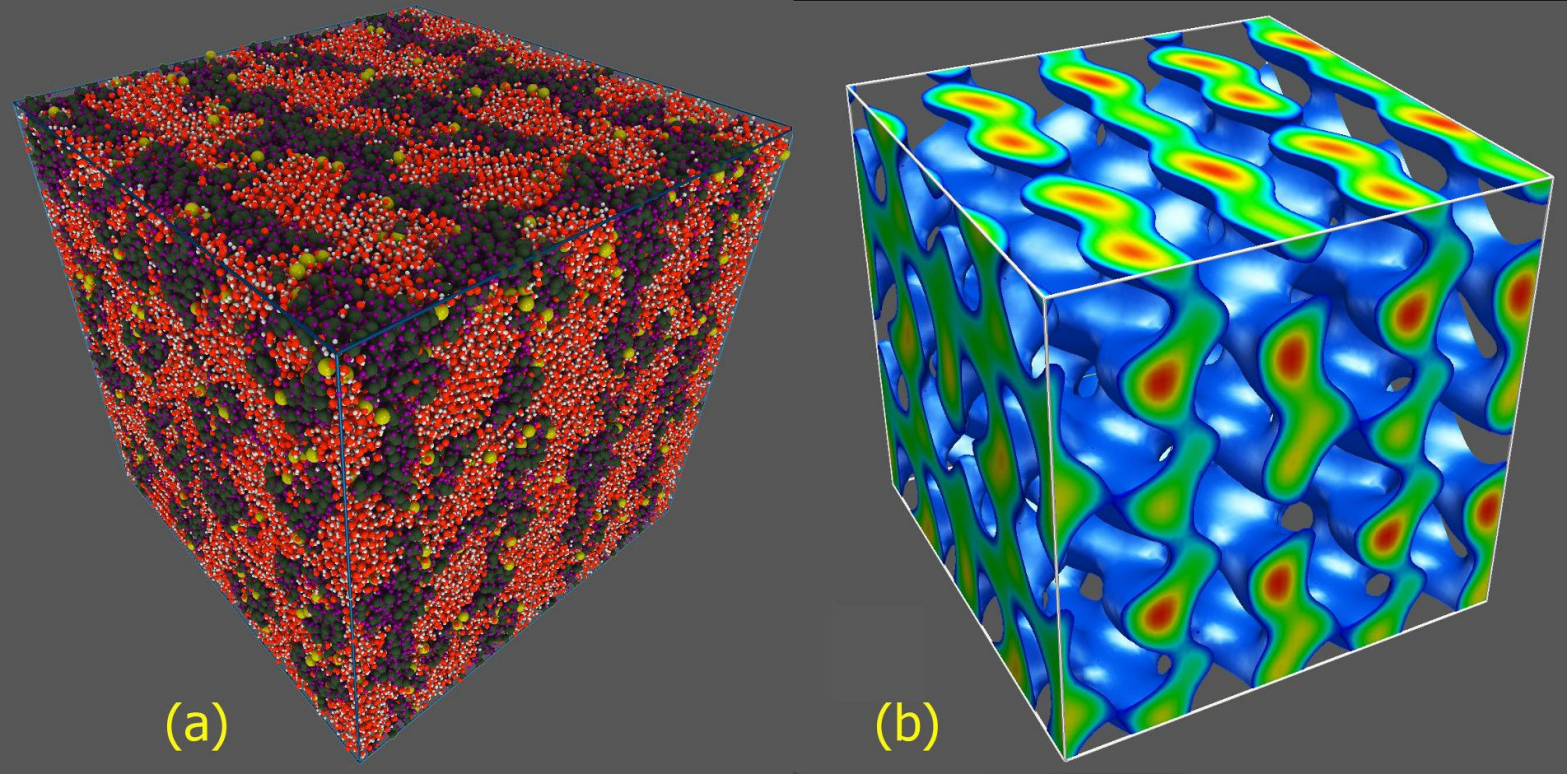
(a) Atomistic representation of the 524,864-atom system and (b) the isodensity surface that demonstrates the distribution of water oxygen atoms in the same system.

The convenient approach to visualize the spatially complex internal structure within the simulated volume is the use of a surface of constant atomic density (isodensity surface) instead of atomistic representation. As an example, we show in Figure 6 the isodensity surface that demonstrates the distribution of water oxygens in the 524,864-atom system at λ = 10. The isodensity surface was defined as ρ_O_/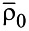 = 1/2, where 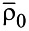 is the average density of water oxygens in the system. As seen, the 3D distribution of the hydrophilic subphase most closely resembles that which is typical for DD structure (Figure 5).

The main result of our large-scale atomistic simulations is the observation that virtually all hydrophilic channels are connected to each other even at relatively low water content in the system. In fact, these channels represent conductive nano-wires which should be responsible for the ionic transport during the operation of a fuel cell. From a global point of view, the channels can be considered as a spatial continuous network organized in an irregular ordered pattern, which is largely similar to that formed by the minority phase of the double-diamond structure. The existence of such a specific structural organization can explain in part the surprisingly high ionic conductivity of the hydrated Nafion membrane.

#### Ion pairing

The ionic conductivity is to a large extent determined by electrostatic interactions and directly related to ion pairing. The accepted mechanism of the ionic conductivity involves the mobile ions moving from one polymer chain to another by a chain-flexing process called segmental motion [77]. To clarify the issue of whether the driving force for dissociation (association) of positively/negatively charged ions, H_3_O^+^ and SO_3_^−^, is dominantly of energetic or entropic nature, we examined the temperature dependence of pair correlation functions (PCFs) *g*_+–_(*r*) and the corresponding potential of mean force *W*_+–_(*r*) = –*k*_B_*T*ln*g*_+–_(*r*). The value of *W*_+–_(*r*) is identified with the free energy *F* as a function of interionic separation *r* at a given temperature *T*. The results discussed in this subsection were obtained from the simulations of our smallest (65,608-atom) system at a hydration level of 10, and the temperature ranged from 200 K to 500 K.

**Figure 7:**
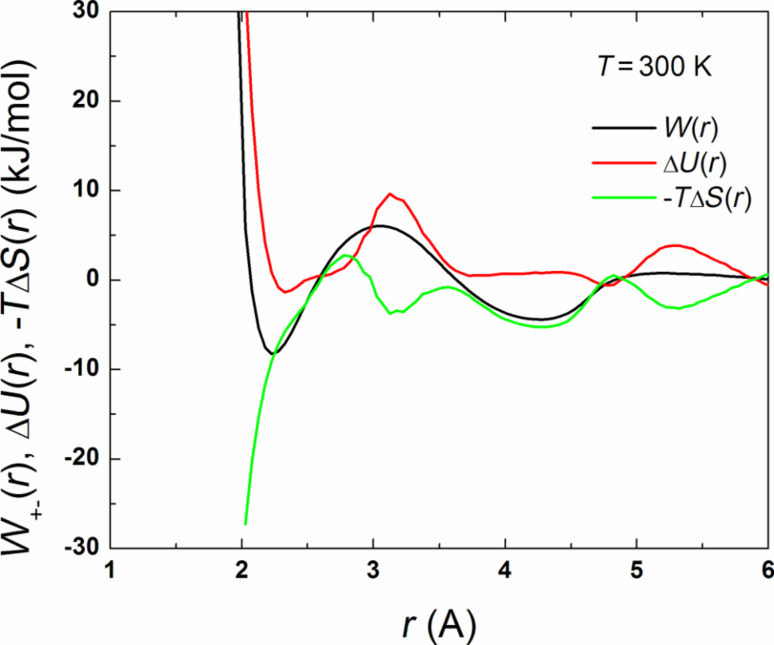
The potential of mean force, *W*_+–_(*r*), and the energetic and entropic contributions, Δ*U* and –*T*Δ*S*, to *W*_+–_(*r*) as a function of the SO_3_^−^–H_3_O^+^ separation at *T* = 300 K.

From the analysis of PCFs we concluded that the ion association is seen to decrease with increasing temperature but a quite strong ion pairing is observed even at *T* = 500 K. On the other hand, no complete clustering is visible even at the lowest temperature considered. It was found that the *W*_+–_(*r*) function demonstrates a rather typical behavior well known for electrolyte solutions: there are one or two deep minima at the distance corresponding to associated sulfonate/hydronium ion pairs SO_3_^−^·H_3_O^+^ and solvent-separated ion pairs as well as a shallow third minimum at longer distances, probably due to a second solvation shell for the ions (Figure 7). As the temperature is subsequently increased, the minimum for the contact ion pairs and solvent-separated ion pairs becomes deeper. More importantly, the main contribution to the free energy of contact ion pairs seems to be dominated by the entropy gain, not the potential energy contribution. To demonstrate this, we performed a thermodynamic decomposition of the potential of mean force.

If the standard state for free energy is defined as that for the infinitely separated cation and anion, *r*→∞, then one can write

**Figure 8:**
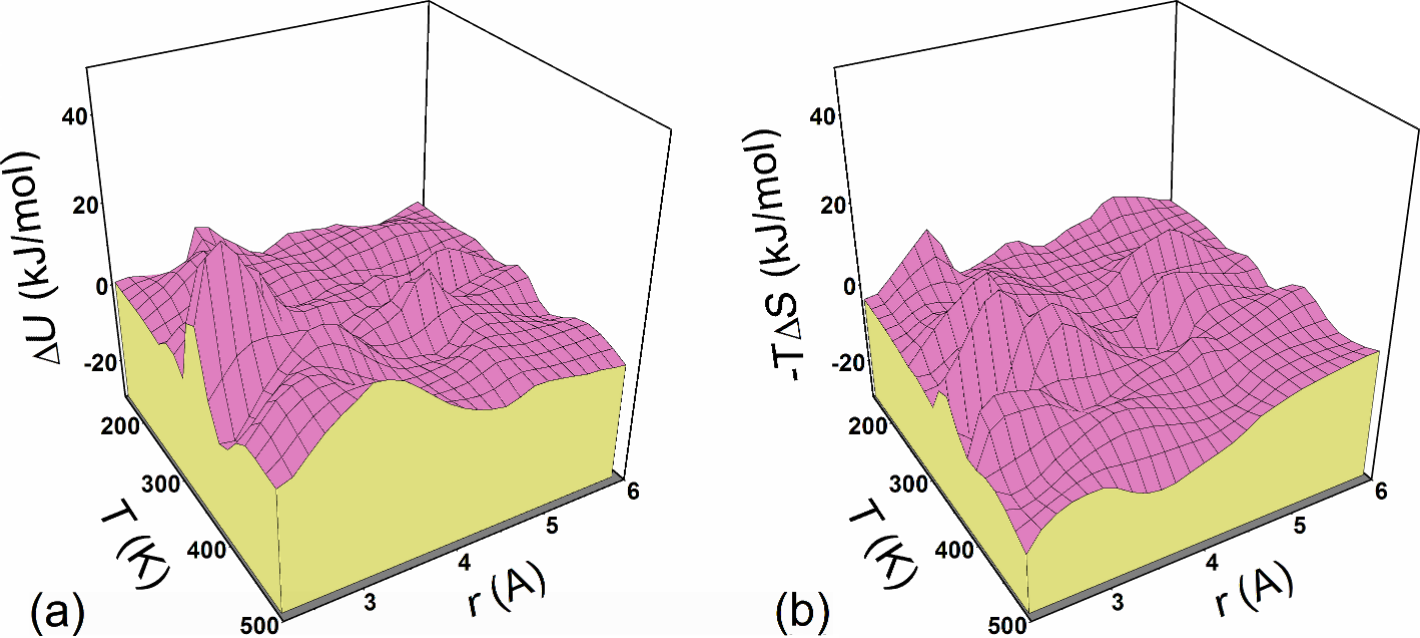
The surfaces (a) Δ*U*(*r*,*T*) and (b) –*T*Δ*S*(*r*,*T*) calculated for the region 2.2 < *r* < 6 Å via the separation of the potential of mean force *W*_+–_(*r*).

[1]
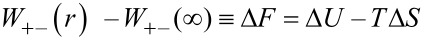


where *W*_+–_(∞) = 0 and Δ*S* = –∂*W*_+–_(*r*)/∂*T*. The entropic part Δ*S* of free energy for different distances and temperatures was calculated numerically using B-splines constructed for the set of the PCFs. Having Δ*S*, one can find Δ*U* from Equation 1. An example demonstrating the behavior of the energetic and entropic contributions, Δ*U* and –*T*Δ*S*, is presented in Figure 7 for *T* = 300 K. Figure 8 shows the surfaces Δ*U*(*r*,*T*) and –*T*Δ*S*(*r*,*T*) calculated for the regions 2.2 < *r* < 6 Å via the separation of the potential of mean force.

As seen, the behavior of the contributions to *W*_+–_(*r*) is rather complex, depending on the regions of *r* and *T*. The energetic contribution acts against squeezing charged groups together below the Lennard–Jones contact distance, as expected. On the other hand, in the region of short interionic distances, including the region of the first minimum of *W*_+–_(*r*), the entropic contribution to the free energy is negative. As the distance increases, the energy appears to weakly prevent the formation of ion pairs, while the entropy gain remains to be the main driving force for contact pairs.

It is instructive to trace the temperature dependence of the energetic and entropic contributions for some specific interion separations, in particular, for those corresponding to the main minimum of *W*_+–_(*r*). For associated ion states (at *r* ≈ 2.28 Å), we see from Figure 8 that the entropic part of free energy demonstrates a general tendency to increase and thus leads to a decrease in Δ*F* as the temperature is increased, while the energetic contribution changes weakly with temperature (except the region located between about 300 and about 400 K, where both Δ*U* and –*T*Δ*S* display oppositely directed oscillations). The superposition of Δ*U* and –*T*Δ*S* results in a monotonous decrease in Δ*F* which appears to be a nearly linear function of temperature.

From the data presented above we can conclude that the change in ion association observed in the system is largely an entropic effect. Ion pairing, which takes place with decreasing temperature, leads to a decrease in entropy and to a corresponding increase in free energy for the entire system. One part of the explanation of this effect follows from loss of low-frequency modes due to ion association. Because these modes are mainly responsible for transport processes, we can expect that the entropic effects would play an important role in the interpretation of the features related to ionic conductivity. Also, it should be noted that an increase in temperature favors the disruption of water-ion contacts, thus leading to an additional increase in the total system entropy.

It is worthwhile to compare the effects of ion pairing observed for polyelectrolytes and for electrolyte solutions, including polymer electrolytes, in which both cations and anions are mobile. A particularly interesting feature of this phenomenon in the case of electrolyte solutions is evidence that pairing increases with increasing temperature [78–79]. It has been suggested (e.g., [80–81]) and shown by computer simulation [82–83] that this effect might occur because more entropy is available to ion pairs than to dissociated ions, leading to an entropically driven temperature dependent contribution to the binding free energy. For the ionomeric system studied here we observe an opposite effect of the temperature, the ion pairing decreases with increasing temperature, although the entropic effect is dominant. Presumably, the different behavior of these systems arises from the mobility of anions in polyelectrolytes, including hydrated Nafion, is strongly restricted.

In addition to the thermodynamic properties, it is instructive to discuss spectral densities of the hindered translational motions of cations. The spectral density is defined by the Fourier transform of the velocity autocorrelation function (VACF)

**Figure 9:**
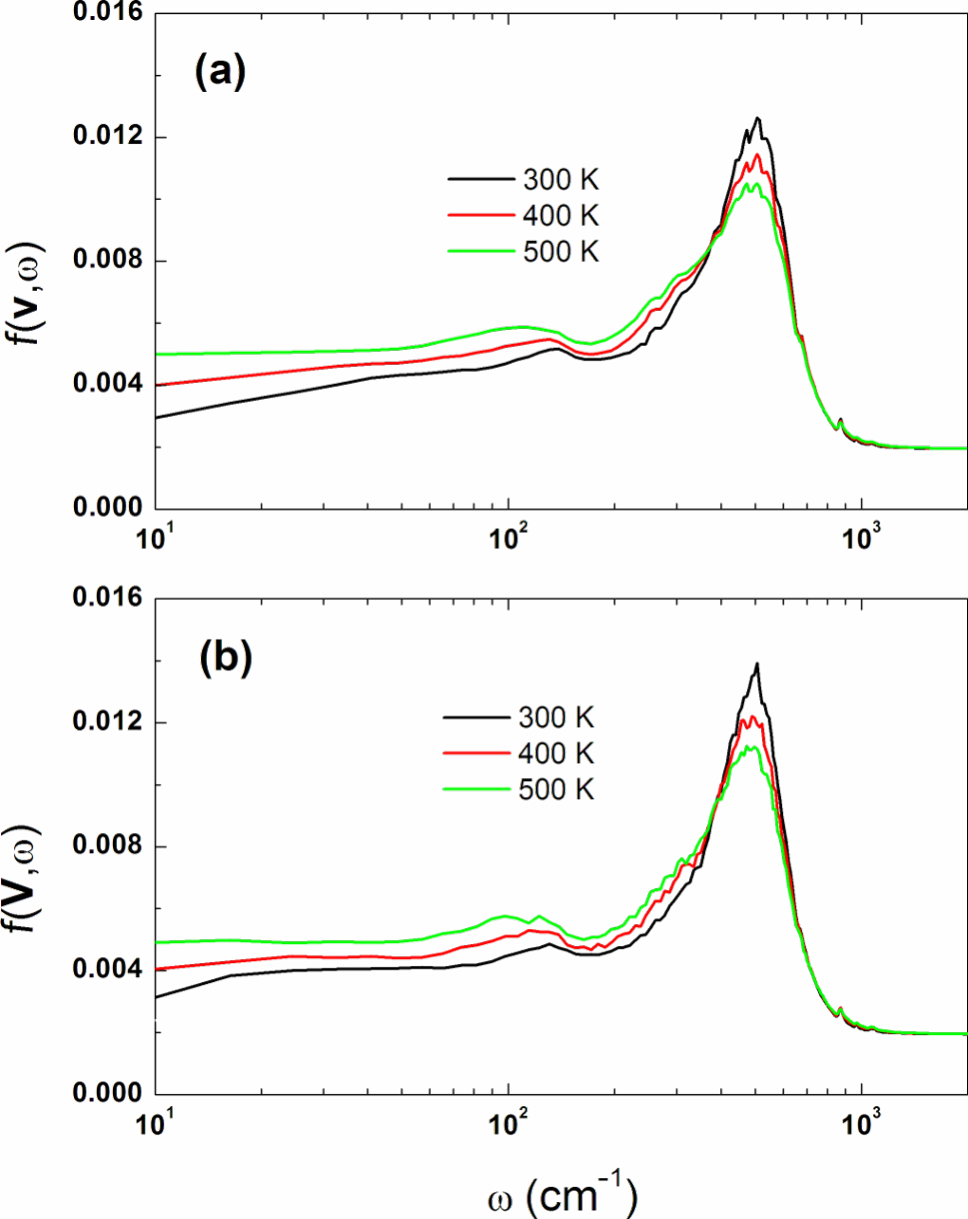
Spectral densities of (a) the hindered translational motions of individual cations and (b) the collective hindered translational motions of the cations calculated at a few temperatures indicated in the figure.

[2]
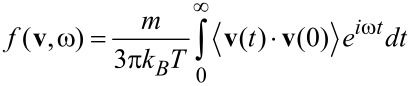


where *m* is the mass of the cation, ω is the frequency, and the factor before the integration sign is chosen so that 
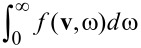
= 1. Also, using the collective VACF 
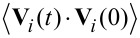
, where 
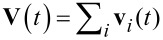
 and **v***_i_*(*t*) is the center-of-mass velocity of an individual cation at time *t*, we calculated the spectral density of the collective hindered translational motions, *f*(**V**,ω), which is directly related to the frequency-dependent ionic conductivity. These functions are presented in Figure 9 for frequencies ω up to 2000 cm^−1^ at three different temperatures.

The results show similar but not identical behavior for *f*(**v**,ω) and *f*(**V**,ω). First, we note that any vibratory component present in the particle motions would appear in spectral densities as a peak away from the origin. These features clearly manifest themselves in the simulated results, which indicate that the motion of the cations has both a vibratory and a diffusive component (Figure 9). In general, such behavior is essentially the same for both spectral densities. Of special interest is the peak located in the region between 490 and 510 cm^−1^, which is believed to indicate the fast vibration of the cation due to the formation of ionic bonds between oxygens of the sulfonate groups and the cation. As the temperature is increased, the vibration intensity at this frequency, ω_o_, becomes lower and this is accompanied by broadening the peak toward lower frequencies and by a weak "red shift" in the main peak. As a result, the diffusive component of motion increases. The same is true for the direct-current (dc) conductivity, which is proportional to *f*(**V**,0). From the power spectra shown in Figure 9 we can conclude that, although significant ion association does exist, the cations are still mobile and would contribute to ionic conductivity even at low temperatures. As has been pointed out above, the loss of low-frequency modes due to ion association is largely an entropic effect.

### Quantum molecular dynamics

#### Models and simulation technique

In the simulation of the structure and dynamics of materials at nanoscale, the electron subsystem (in many cases) should be taken into account in an explicit form. Naturally, this requires the solution of the Schrödinger equation to describe the quantum evolution of the system of many nuclei and electrons. This approach is accomplished in quantum molecular dynamics (QMD), which considers in combination the motion of classical (atomic nuclei) and quantum (electrons) particles [84]. The model treats simultaneously the alteration of wave function defining electron density redistribution and the change of coordinates of classical atoms, i.e., the Schrödinger and Newton equations are solved in combination at each time step. The approach consists in the determination of forces affecting atoms "on the fly" from electronic structure calculations based on the first (ab initio) quantum-mechanical principles rather than on empirical potentials. This provides a possibility to observe not only structural properties of materials at classical level but also electronic effects responsible for chemical transformations. However, the QMD method requires computer resources by orders of magnitude larger than those sufficient for typical atomistic MD simulations. In this work, we describe for the first time the results of QMD modeling of microphase separation and ion-conducting channels in a Nafion membrane. To that end, two models were developed.

**Model I.** First of all, we tested whether it is possible to predict the microphase separation of water and Nafion using QMD. For this purpose, a relatively small system consisting of six identical sulfonated Nafion monomers (Figure 1b) and 30 or 60 water molecules (corresponding to a hydration level of 5 or 10) was studied in a cubic box with periodic boundary conditions. The initial distribution of molecules in the box was random.

**Figure 10:**
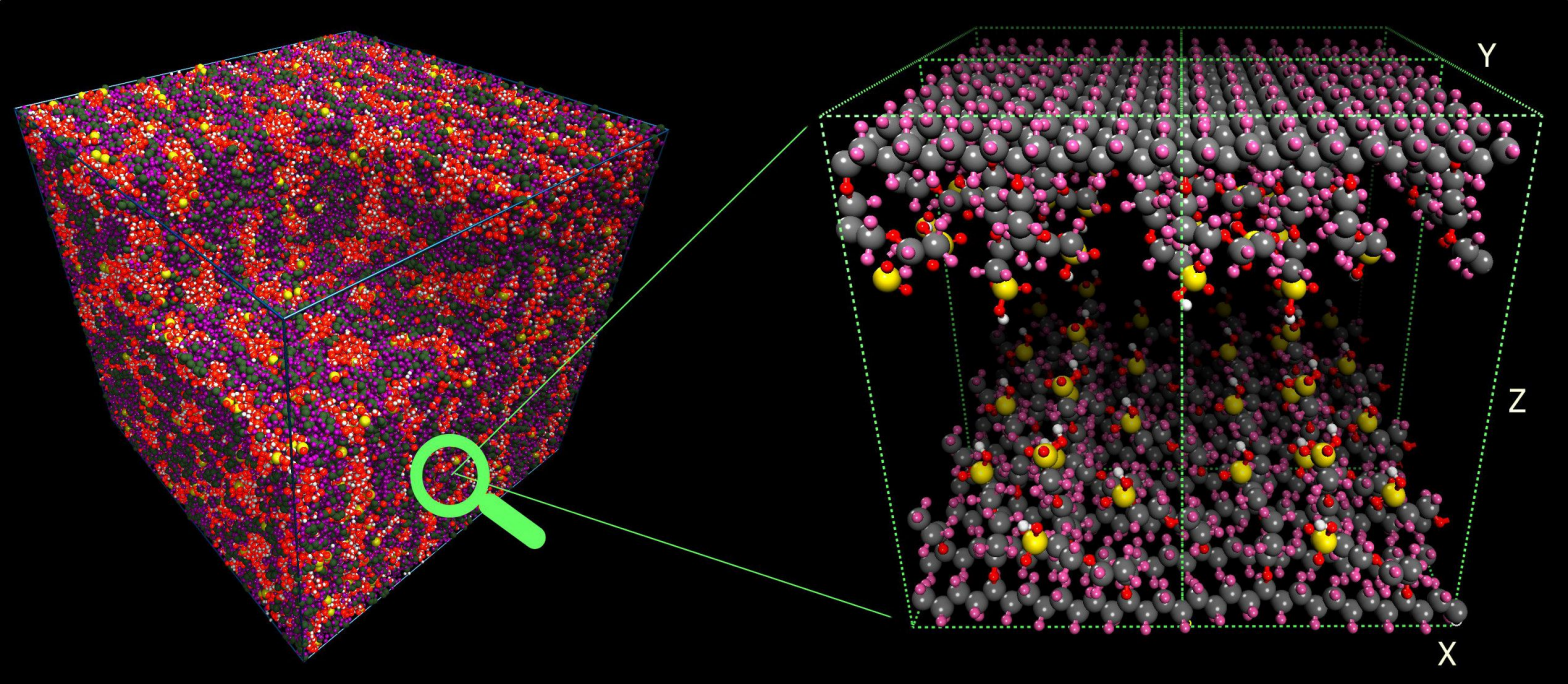
Model of the ion-conducting channel studied by quantum molecular dynamics. The initial configuration is shown. Channel walls are formed by Nafion chains oriented along the *X*-axis. The chains are infinite in the periodic cell. The total number of atoms in the system is 1200. Water molecules filling the channel are omitted for visual clarity. Conventional atom colors are used.

**Model II.** The model of an ion-conducting channel was based on the data obtained from our classical MD simulation at λ = 10 and *T* = 298 K. The channel was modeled as a slit whose two opposite walls separated by a distance of 37.21 Å along the Z-axis were built of eight Nafion strands oriented along the X-axis of a unit simulation cell with periodic boundary conditions (Figure 10). The lateral distance between the neighboring Nafion strands was extracted from atomistic MD simulations and refined later by QMD. The chains are virtually infinite in the periodic cell. Each periodic strand contains two sulfonic acid groups, resulting in sixteen SO_3_H groups per unit cell. The interior of the channel is filled with water molecules, resulting in a 1200-atom system. The dimensions of the rectangular unit cell are 20.46 × 19.91 × 37.21 Å along the X-, Y-, Z-axes, respectively. The thickness of the slit (37.21 Å) adopted in the model corresponds to the average size of the hydrophilic channel estimated from the atomistic MD.

We employed the CP2k package [85] and the so-called "Car–Parrinello-like approach to Born–Oppenheimer MD" recently developed by Kühne et al. [86]. This hybrid quantum mechanical simulation scheme combines Born–Oppenheimer quantum molecular dynamics (BOMD) [84] and Car–Parrinello molecular dynamics (CPMD) [87] within Kohn–Sham electron density functional theory (DFT) [88]. As in the original CPMD, the electronic wave functions are not self-consistently optimized here during the evolution of electrons and ions. However, the fictitious Newtonian dynamics is substituted by a similarly coupled electron-ion MD. Importantly, this computational scheme does not require the use of a fictitious mass parameter, but similar to BOMD, the electrons are kept on the Born–Oppenheimer surface, corresponding to their instantaneous electronic ground state, by means of explicit electronic structure optimization after each MD step [89]. This implies that the time step can be chosen to be as large as the particular ionic resonance limit. The main advantage of this hybrid approach is that the best aspects of both conventional QMD methods, CPMD and BOMD, are exploited simultaneously. From a computational viewpoint, the hybrid CPMD/BOMD scheme proved to be much more efficient than BOMD and CPMD separately, thereby allowing one to increase the size of simulated systems and use rather complex variants of DFT.

We used a recently modified version of the code CP2K/Quickstep [85], which is a numerical implementation of the Gaussian and plane waves (GPW) method [90] based on the Kohn–Sham formulation of DFT. The electronic charge density was expanded in an auxiliary plane-wave basis at the Γ-point up to a kinetic energy cutoff of 280 Ry. The high-precision "molecular-optimized" (MOLOPT) triple basis set with a supplementary set of polarization functions (TZVP-MOLOPT-GTH) [91] was employed. This basis set was specifically optimized to perform accurate DFT-based molecular calculations for a wide range of chemical environments, including gas phase, interfaces, and condensed phase [91]. The Kohn–Sham orbitals were expanded in contracted Gaussian basis sets. Core electrons were removed by the introduction of norm conserving pseudopotentials developed by Goedecker, Teter and Hutter (GTH) [92]. The exchange–correlation potential was approximated by the PBE-D functional supplemented by a damped interatomic potential to account for van der Waals interactions [93]. This approach turned out to be reasonably accurate yet computationally affordable for our systems, which are very large. The dispersion correction term was used because it is known that the inclusion of van der Waals interactions systematically improves the density of liquid water [94]. All the technical parameters (γ_D_, *K*, etc.) of the hybrid CPMD/BOMD method were selected as recommended in [95], where static and dynamical properties of liquid water were studied. The QMD computations were performed on 1024 nodes of the supercomputer "Lomonosov".

Given the high computational cost of a QMD simulation, the initial configuration of the ion-conducting channel (Figure 10) was first pre-equilibrated for 10 ns using a classical PCFF force field, followed by a 50-ps equilibration with QMD in the canonical (NVT) ensemble. The integration of the equations of motions for ions was performed using the velocity-Verlet algorithm coupled to a Nose–Hoover chain thermostat on each nuclear degree of freedom. The target temperature for the equilibration was set to 400 K. The temperature was then reduced to 298 K and the simulation was continued for additional 110 ps. After that the productive QMD run was performed for 120 ps at 298 K. This time window is sufficient for studying the charge-transport processes in a system where low energy barriers are effectively washed out by zero-point motion. Because the initial configuration for the QMD simulations of the nanochannel was taken from the classical MD trajectory, it was important to check the stability of the model channel. No strong drift of the structure was observed over most of the 120-ps QMD simulation and therefore the system remains stable.

In the QMD simulations of Model I, the same methodology and protocol as described above were applied, except that the simulation time was 200 ps (after 100 ps of equilibration). Note that a rather long equilibration time (at least 100 ps) is required to achieve an equilibrium content of water–proton complexes (especially Eigen cations) in the system.

#### Segregation of water and perfluorosulfonic acid molecules

**Figure 11:**
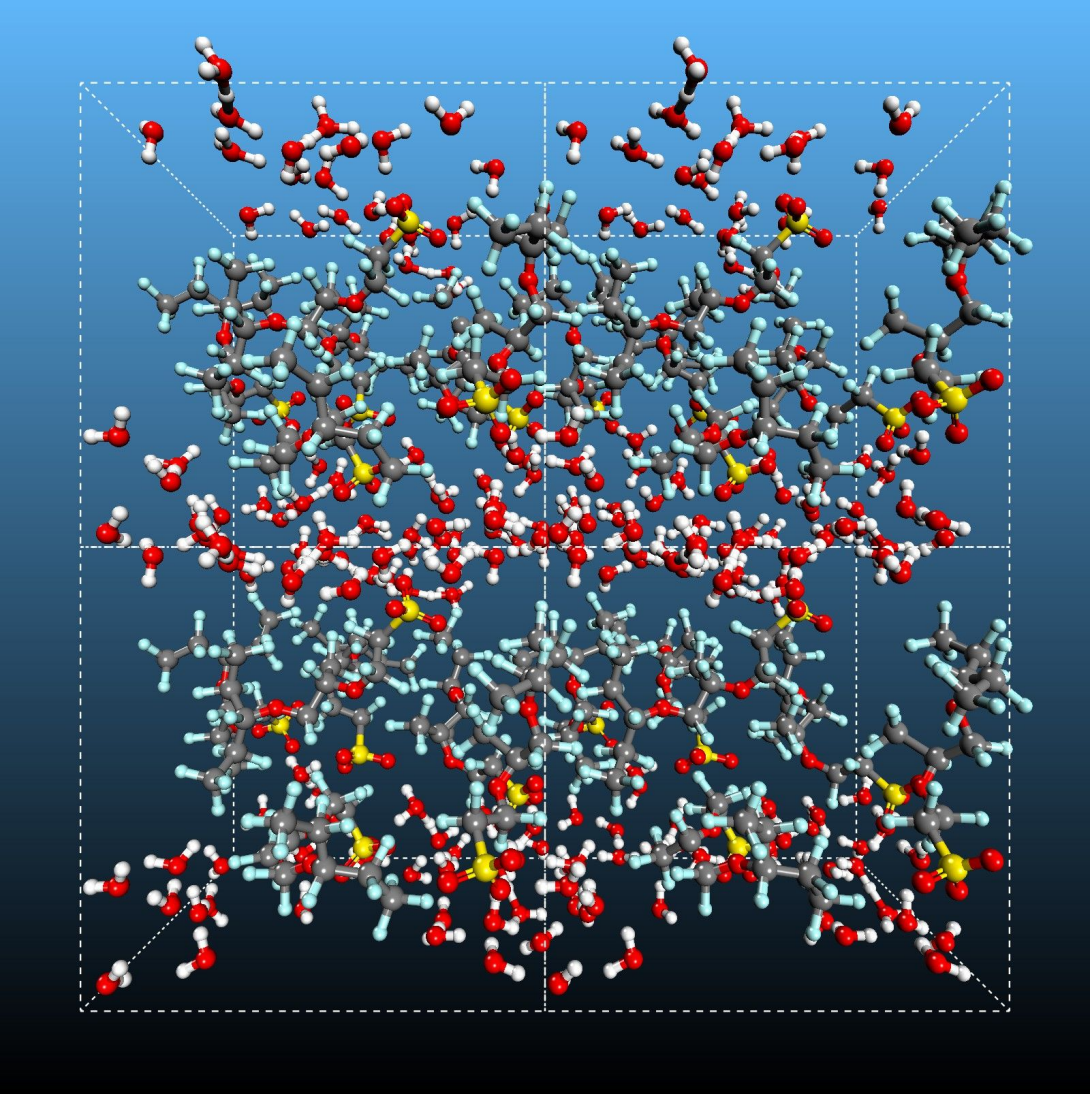
Snapshot of the water-containing Nafion structure obtained after the 200 ps QMD simulation at 298 K and λ = 10. The simulation box is replicated two times along the X- and Z-directions. For clarity, some of the equivalent atoms (not all) across the periodic boundaries are also depicted. Oxygen is shown in red, carbon in gray, fluorine in light blue, hydrogen in white, and sulfur in yellow.

The final molecular configuration obtained for Model I at λ = 10 is presented in Figure 11. As seen, the Teflon backbone and hydrophobic side chains are pushed out of the aqueous medium by the water molecules, while the strongly polar SO_3_^−^ groups bound to the side chains favor contact with water and, as a result, they tend to localize at the interface between the hydrophobic units and water. From examination of various snapshots, it is evident that a lamella-like microphase-separated structure is formed in this small system. Thus we can conclude that even though the system is not large enough to investigate the nanoscale morphology, a hydrophilic/hydrophobic segregation indeed occurs in the hydrated Nafion. Of course, the system size is also too small to develop more complex micro-segregated morphologies like bicontinuous superstructure. Actually, we observe the formation of a hydrophilic channel in the system. Also, the formation of both hydronium ions and more complex hydrated proton clusters is clearly seen (Figure 11). This issue is discussed in more detail in the next subsection.

**Figure 12:**
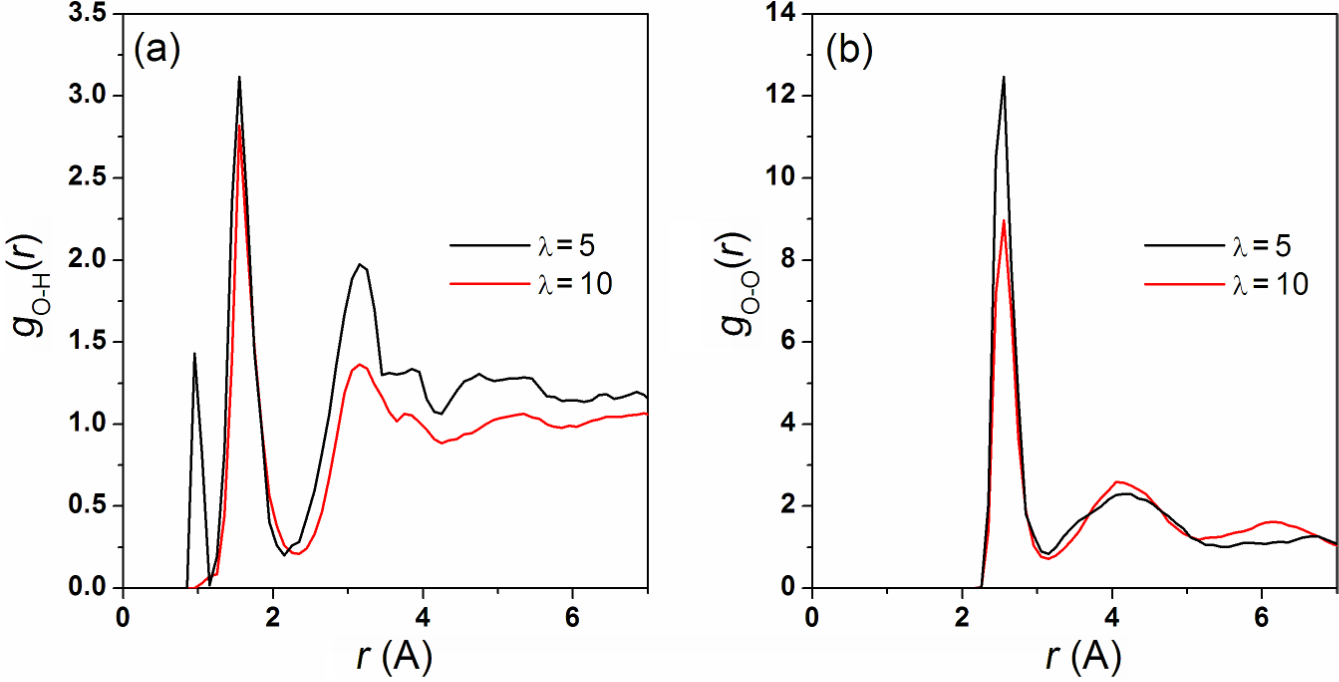
(a) Pair correlation functions, *g*_OH_(*r*), for the oxygen atoms of the SO_3_ groups and any proton, at λ = 5 and 10. (b) Pair correlation functions, *g*_OO_(*r*), for the oxygen atoms of H_2_O and proton–water complexes, at λ = 5 and 10.

Additionally we analyzed pair correlation functions (PCFs). Figure 12a shows PCF *g*_OH_(*r*), where O indicates the oxygen atom belonging to the SO_3_ groups and H denotes any hydrogen. At the hydration level λ = 5, the first sharp peak located at *r* ≈ 1.01 Å corresponds to the O–H bond distance in non-dissociated SO_3_H. This means that at low water loading, not all SO_3_H groups are ionized. At the same time, it should be stressed that perfluorosulfonic acids require only three water molecules to exhibit spontaneous proton dissociation [57]. At the higher hydration level, λ = 10, practically all protons are dissociated and this "valence" peak is not visible. The main peak in *g*_OH_(*r*) is associated with the first coordination shell formed by water molecules and positively charged ions around the SO_3_ groups, giving an average first solvation shell size of approximately 3.5 water molecules.

In Figure 12b we show the *g*_OO_(*r*) function calculated for the oxygen atoms of water and the oxygen atoms of charged water-proton complexes H^+^(H_2_O)*_n_* (for the definition of H^+^(H_2_O)*_n_*, see the next section). It has the first sharp peak around 2.5 Å, which is associated with the formation of hydrated proton complexes. The remarkable intensity of this peak is an indication that water molecules are considerably localized near these complexes. The area under the first peak corresponds to a coordination number of about 3. The differences in *g*_OO_(*r*) between the systems at λ = 5 and 10 are comparatively minor: no significant difference on the peak positions of the PCFs occurs. Nevertheless, the second and third coordination spheres become more pronounced when λ is increased.

#### Proton solvation in hydrophilic channel

In simulating the model of an ion conducting nanochannel (Model II), our main goal was to reveal the details of the structural and dynamic mechanism of charge transfer. The statistical analysis of the 120-ps trajectories obtained from ab initio MD simulations allows us to establish some general features of the mechanism responsible for proton solvation and transport inside the hydrophilic channels of PEMs.

The sulfonic acid groups of Nafion in aqueous surrounding release protons, which bind to water molecules to give hydronium ions. Once hydronium ions are formed, they do not stay alone, because they develop more complex hydrated proton forms with nearby water molecules in continuously exchanging configurations. These configurations include, e.g., the Zundel cation H_5_O_2_^+^, the Eigen cation (a triply solvated hydronium ion (H_2_O)_4_H^+^ or H_3_O^+^·(H_2_O)_3_), and other more complex solvation structures H^+^(H_2_O)*_n_* with *n* > 4 [96–100]. In the Zundel cation, the proton mainly resides in between two water molecules, H_2_O^…^H^+…^OH_2_. The Eigen cation consists of a hydronium core symmetrically solvated by three additional water molecules [96–100]. In liquid water or other hydrogen bonded liquids, the description of the proton solvation is usually limited by the Zundel cation and the Eigen cation. The difference between potential energy of these cations is very small (ca. 2–3 kcal/mol); as a result, these two solvation forms can interconvert on the femto- to picosecond time scale [97]. While the proton transport mechanism is believed to involve the inter-conversion between these cations [97], the details of the solvation process and aqueous proton transfer are still unknown for ionomer membranes.

**Figure 13:**
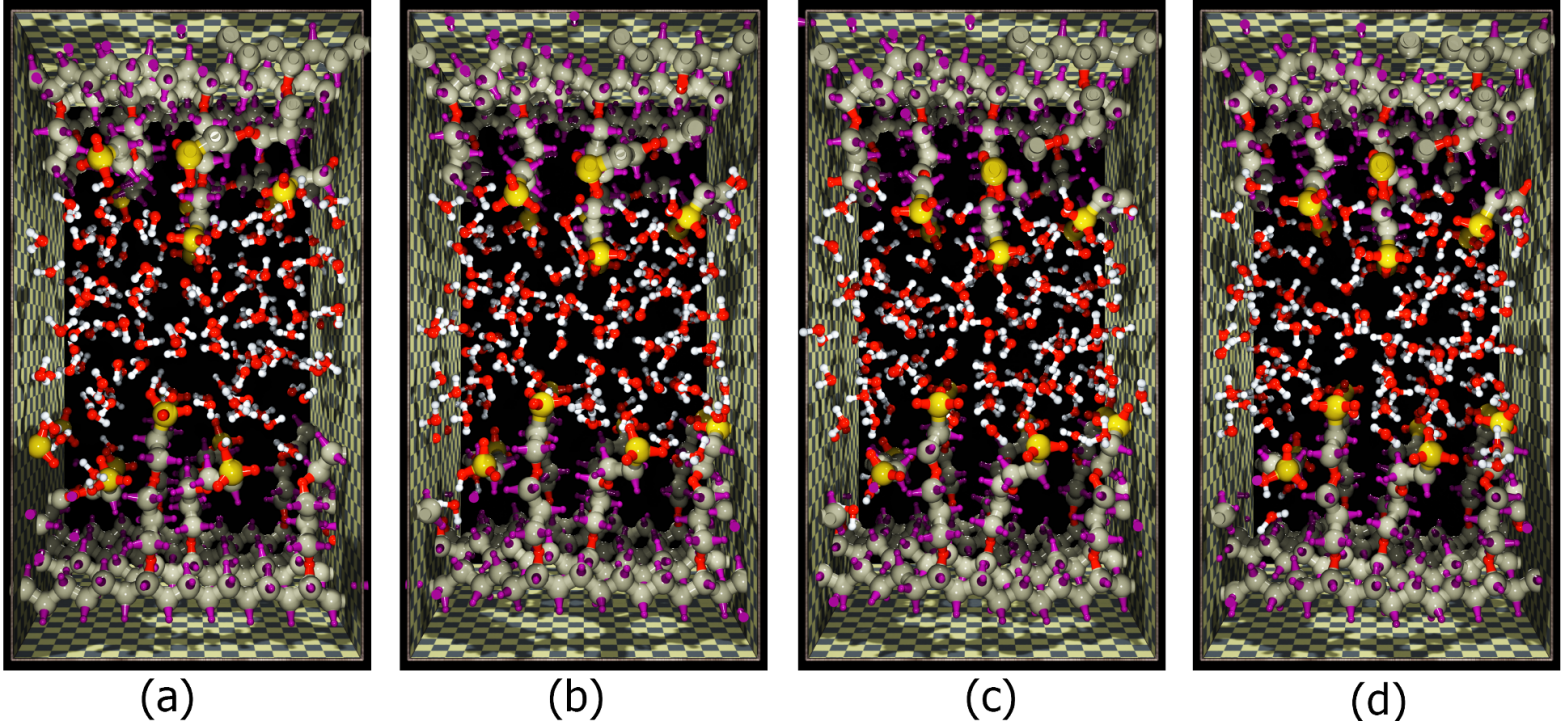
Sequence of snapshots from the QMD simulation of the ion-conducting nanochannel at different time points, starting with the initial configuration (a).

In order to identify water–proton complexes H^+^(H_2_O)*_n_* with *n* = 1–4, the hydronium oxygens were first selected as those closest to the centre of excess positive charges. After that their three hydrogens were found by a shortest-distance criterion. Finally, the complexes were completed by adding the closest water molecules. At each time step the hydrated proton forms were constructed anew. Snapshots of the simulated system clearly demonstrate the formation of various proton complexes (Figure 13).

The QMD simulations showed that the presence of water in the model nanochannel causes dissociation of –SO_3_H units resulting in the formation of the SO_3_^−^·H_3_O^+^ ion pairs. The dissociation and transfer of the hydrogen ion to the aqueous medium occur very fast, typically within 0.1–0.2 ps. Due to the further solvation, the ion pairs transform into shared proton complexes SO_3_^−^·H_3_O^+^·(H_2_O). The Zundel cations are then formed via the proton transfer reaction H_3_O^+^ + H_2_O → H_5_O_2_^+^. At the next step, the topological defects in the hydrogen bond network occur in the form of Zundel–Zundel transformations. Also, the solvated hydronium complexes in the form of the Eigen cations exist as an intermediate state in Zundel–Eigen–Zundel proton exchange.

The hydronium and the nearest water molecules constantly interchange the proton within a very short time. In this way, the excess proton rattles between the oxygen atoms of two neighboring water molecules. In other words, the proton is transferred temporarily from a water molecule to its neighboring molecule by hopping, but then returns to its original location. It is clear that this process will not contribute significantly to the actual diffusion of protons in water phase. This picture is in agreement with both experimental observation [101] and theoretical work [102] which predicts that proton transfer along the hydrogen bond network should be essentially barrierless. Also, the proton may rattle between water molecules and the SO_3_^−^ units similar to the rapid hopping between H_3_O^+^ and H_2_O.

We calculated the average relative content (per SO_3_^−^ group) of different hydrated proton complexes [A]/[SO_3_^−^], where [A] denotes the concentration of the species H^+^(H_2_O)*_n_* and [SO_3_^−^] is the concentration of SO_3_^−^ groups. The results are shown in Table 1.

**Table 1:** Average relative content of different hydrated proton complexes.

ion	H_3_O^+^	H_5_O_2_^+^	H_7_O_3_^+^	H_9_O_4_^+^	H_3_O^+^·(H_2_O)_3_

[ion]/[SO_3_^−^]	0.664	0.294	0.004	0.000	0.438

It is seen that the hydronium, Zundel and Eigen ions dominate in the system, while the H_7_O_3_^+^ and H_9_O_4_^+^ complexes are very rare.

**Figure 14:**
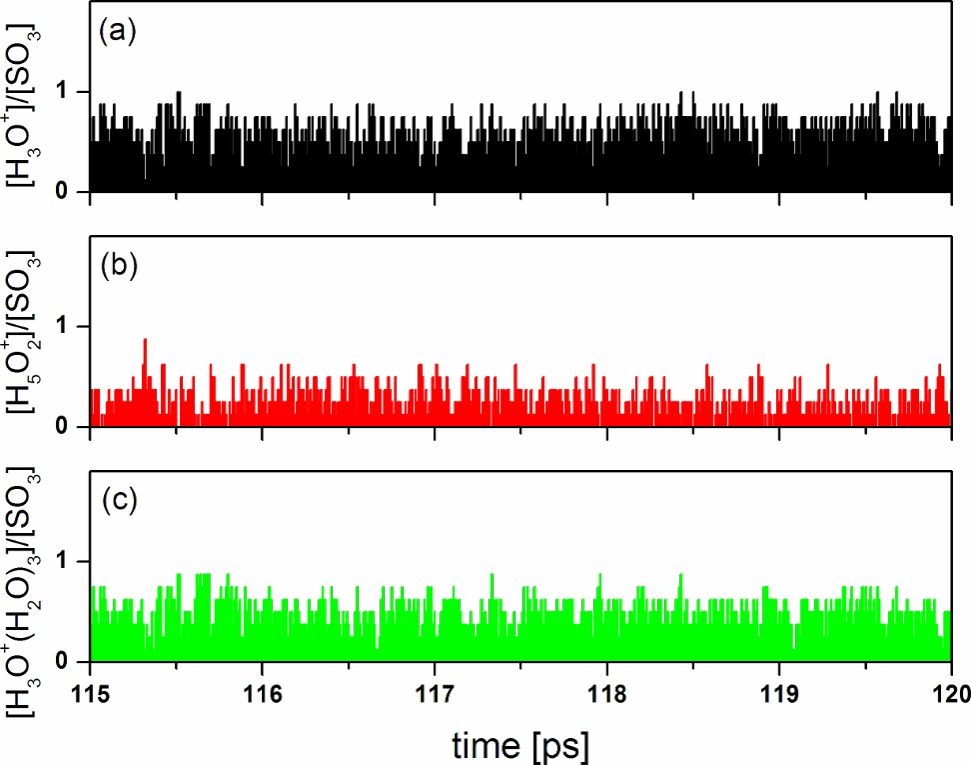
A 5-ps section of a QMD trajectory showing the change in the relative content of different hydrated proton complexes at λ = 10. This short time section is chosen for visibility, but the behavior shown here is typical of any time section from the same trajectory.

**Figure 15:**
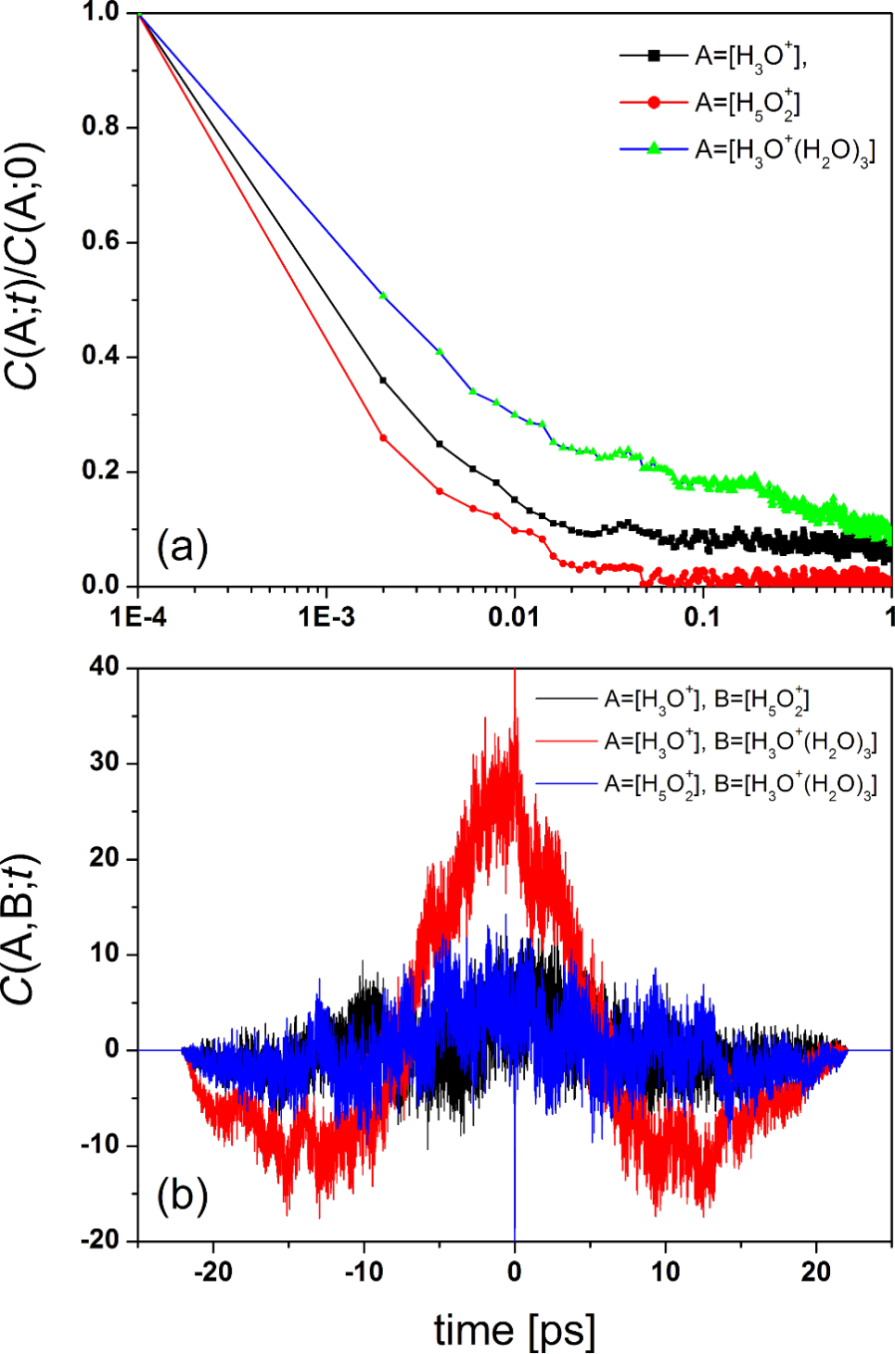
(a) Normalized time autocorrelation functions for the processes [A](*t*), where A denotes H_3_O^+^, H_5_O_2_^+^, and H_3_O^+^·(H_2_O)_3_, at λ = 10. (b) Time-dependent cross-correlation functions (in arbitrary units) characterizing the formation of different pairs of hydrated proton complexes at λ = 10.

In Figure 14 we present the relative content of hydrated proton complexes [A], A = H_3_O^+^, H_5_O_2_^+^, H_3_O^+^·(H_2_O)_3_, as a function of simulation time. The time autocorrelation functions, *C*(A;*t*), calculated for these processes and the time-dependent cross-correlation functions, *C*(A,B;*t*), characterizing the correlation between three different pairs of the same complexes are shown in Figure 15. It is notable that the *C*(A;*t*) correlation functions exhibit an exponential decay at very short time (≈10 fs). The estimated relaxation time τ_h_ associated with the formation of hydronium ions is greater than the relaxation time τ_Z_ found for Zundel ions, but is considerably less than the relaxation time τ_E_ found for Eigen ions. This result suggests that the proton exchange between hydronium and water molecules is a relatively fast process as compared to the formation of both hydronium and Eigen ions. An analogous conclusion can be drawn from the data shown in Figure 15b for the cross-correlation functions. The reversible transition H_3_O^+^ + 3H_2_O ↔ H_3_O^+^·(H_2_O)_3_ is a strongly correlated process, as can be expected, while the mutual transformations of hydronioum/Zundel ions (reversible transition H_3_O^+^ + H_2_O ↔ H_5_O_2_^+^) and Zundel/Eigen ions (reversible transition H_5_O_2_^+^ + 2H_2_O ↔ H_3_O^+^·(H_2_O)_3_) are less correlated. Note that proton transmission becomes possible only when the surrounding water molecules rearrange at particular points in time to enable the Zundel cation and at other times the Eigen cation configuration.

The widely accepted view is that the proton transfer process can generally be described as the sequential transformation hydronium–Zundel–hydronium (h–Z–h) [5–6]. This mechanism is highly analogous to the Eigen–Zundel–Eigen (E–Z–E) transformation model of proton transfer in water [97,102]. For hydrated polymeric membranes, however, the situation may in principle be different. In this case, one may wonder what influence the presence of SO_3_^−^ groups has on the proton hydration and transfer. The key difference between bulk water and the membrane is that in membrane, there is a high surface density of SO_3_^−^ groups which are located at the water/Nafion interface (cf. Figure 11 and Figure 13). Thus, the nanochannels can be understood as high surface charge density pores where the passage of charge should be affected by Donnan exclusion effects, whose exact role and mechanism, however, is still being debated (for more details, see [103]). Due to the fact that protons may be trapped by negatively charged SO_3_^−^ groups, they remain for a relatively long time at the interface instead of being released into the bulk. Hydronium cations formed in this interfacial region are closer to the surface than water molecules so that they can more or less easily be transformed into Zundel ions at the hydronium/water interface, but not into Eigen ions. The results shown in Figure 15b directly confirm this behavior.

It follows from the analysis of PCFs and snapshots that the configuration in which a hydronium ion is hydrogen bonded to oxygens of two SO_3_^−^ groups is frequently found in the system. Due to steric hindrance, the accessibility of the hydronium with respect to the surrounding water molecules decreases in this configuration. Such a steric hindrance makes it difficult to form a hydrogen bond between the hydronium and neighboring water molecules. As a result, a proton is detached from a SO_3_H group, transferred temporarily from this group to its neighboring deprotonated group SO_3_^−^ by hopping (assisted or not assisted by surrounding water molecules), but then can return to its original location. In a sense, this behavior is reminiscent of the "cage effect" in classical liquids, where a particle can be trapped inside a virtual cage formed by its neighbors for some time, before it can escape from this cage and then diffuse in the bulk liquid. It may well be that this is one of the reasons why the relaxation time τ_h_ is greater than τ_Z_ (cf. Figure 15).

Since the SO_3_^−^ groups are close to each other in the water channel, free protons can reside in between these groups, thereby decreasing the effective degree of dissociation. We call this dissociation mechanism "abnormal dissociation". Schematically, the process can be represented as follows: SO_3_H + SO_3_^−^ ↔ SO_3_^−^ + SO_3_H. It is clear that the "abnormal dissociation" will reduce the number of proton participating in the protonation of water. Naturally, in these circumstances, we should speak about an "effective dissociation" that generally is lower as compared to an expected 100% dissociation. In practice, however, a significant decrease in the degree of dissociation can be expected only for a very narrow channel, at low water loading.

Thus, from the results of the QMD simulations, one can expect that for our model nanochannel, proton transfer through Zundel–Eigen–Zundel (Z–E–Z) transformations, which feature importantly in the transfer of the protons at high water content [97,104], should dominate over the transfer through Zundel–Zundel (Z–Z) transformations. On the other hand, in agreement with the scenario proposed by Paddison et al. [105] and Idupulapati et al. [106], the dominant transport mechanism consisting of sequences Z–Z transformations can be favorable at very low water content when the channel diameter is very small, comparable to the size of the Eigen cation.

#### Proton transport and the Grotthuss mechanism

There are several hypothetical models that describe proton transport in aqueous media. The most known and widely discussed model that explains the reasons of abnormally fast diffusion of the proton in water was formulated by Grotthuss more than 200 years ago [107–110]. This model implies that chains of dipoles can explain the transport of charge in water. For a long time, the Grotthuss mechanism (also called the *hop-and-turn* or *relay* mechanism) was no more than an elegant hypothesis. In 2005, Nibbering and colleagues [109] found that hydrogen ions are indeed transmitted very efficiently through water, as predicted by the Grotthuss model [107]. It should be noted that this experimental study become possible by using a unique technique based on ultrashort laser flashes, that enabled the determination of the jump-like proton transmission from acids via water to bases in time steps of 150 femtoseconds. Very recently, Kulig and Agmon also presented the experimental evidence of the Grotthuss mechanism, using a "clusters-in-liquid" approach for calculating the infrared spectrum from any set of charges in bulk water and water clusters [100]. It should be borne in mind that in general, the mobility of protons in aqueous environments is determined by a combination of the two contributions: a fast Grotthuss-type proton hopping and the much more slow hydrodynamic diffusion of protonated water clusters [110]. It cannot be said, however, that the problem related to the Grotthuss mechanism has found a unified solution. The subject has given rise to renewed interest particularly in view of the growing role played by ab-initio molecular dynamics methods applied to the transport of excess protons in water and water solutions (see, e.g., [102,111–118]).

The Grotthuss proton-transfer process implies that an excess proton moves through the hydrogen bond network of water molecules through the formation/cleavage of O–H bonds when the Zundel or Eigen cation can form. From the chemical viewpoint, such process corresponds to heterolytically dissociating and reforming individual H_2_O molecules. Of course, all molecules in liquid phase are also involved in normal thermal motion. Therefore the resulting charge migration should be a superposition of two alternating motion motifs: (i) usual "slow" (vehicle or Stokes) diffusion of a proton within the hydronium ion, which behaves as a transport container and (ii) the fast proton hopping far along the network of hydrogen bonds between neighboring water molecules. This means that the dynamics of charge transfer should involve at least two distinctive characteristic times and corresponding to them two characteristic spatial scales related to one or another motion motif.

To verify this hypothesis, we calculated the autocorrelation portion *G_s_*(**r**,*t*) of the van Hove space–time correlation function *G*(**r**,*t*) [119], which is a fundamental property of liquid structure and in practice results from the Fourier transform of the so-called intermediate scattering function obtained experimentally from incoherent neutron scattering. The *G_s_*(**r**,*t*) function provides a probability to detect a particle at moment *t* in a space point **r** on condition that it was in the origin of coordinates at *t* = 0. However, unlike common computations, we monitored positive charge movement rather than particle position. This methodology is similar to an "identity concept" proposed by Agmon and coworkers [120]. Also, like this work, we did not differentiate between "unproductive" and "productive" (successful) proton transfer. The *G_s_*(**r**,*t*) function defined in this way is relevant for characterizing the heterogeneous dynamics related to proton transfer.

**Figure 16:**
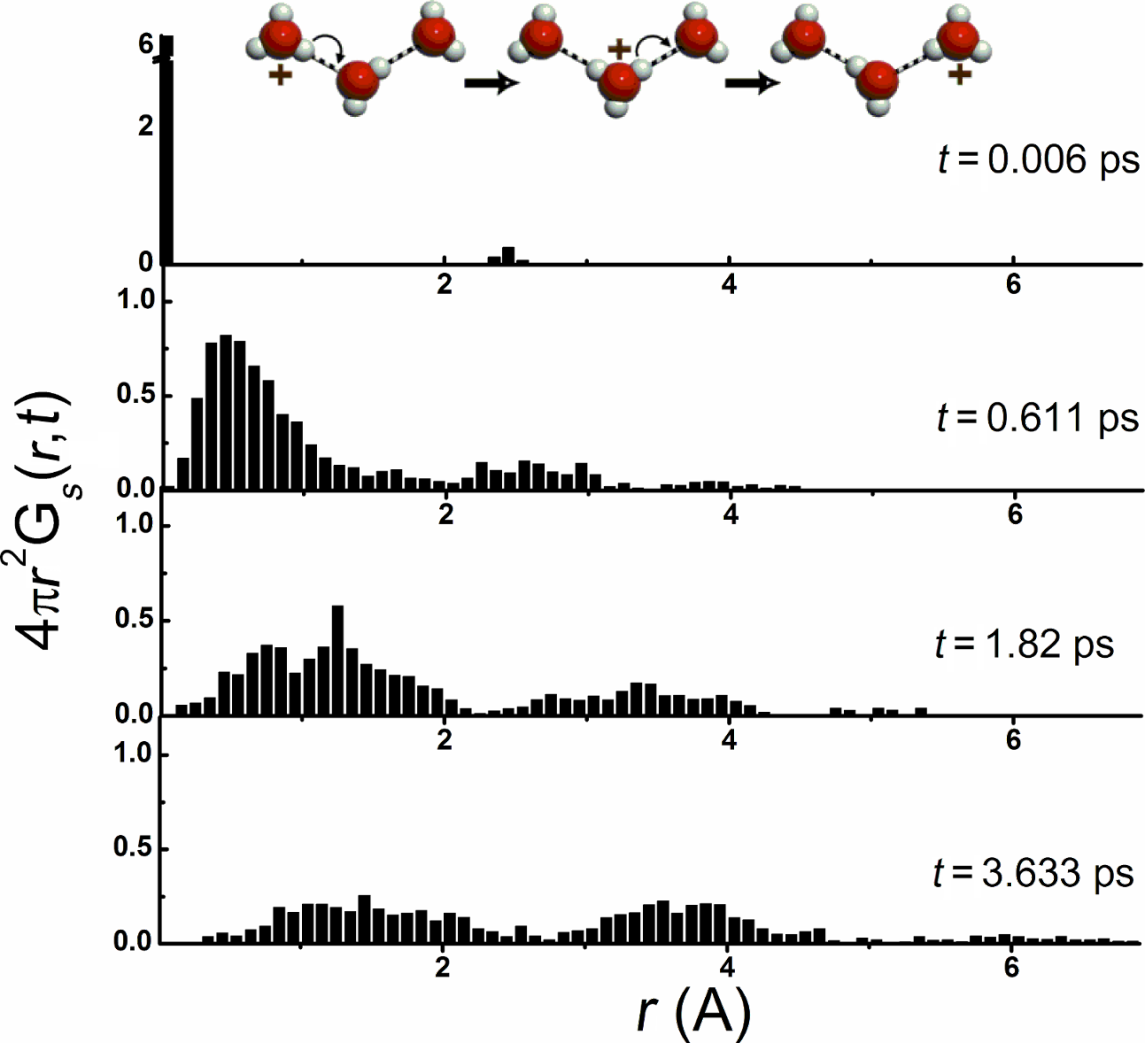
The *G_s_*(**r**,*t*) correlation function is the time-dependent conditional probability density that a particle (charge) moves a distance *r* = |**r**(0) − **r**(*t*)| during time *t*. At *t* = 0, *G_s_*(**r**,0) = δ(*r*), whereas in the long time limit, the system looses memory of the initial configuration and *G_s_*(**r**,0) becomes independent of the distance *r*: 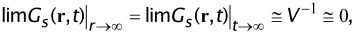 where *V* is the system volume.

The results are shown in Figure 16 for four different time intervals. The sufficiently large extent of generated QMD trajectories and obtained statistics allow one to observe the bimodality of the *G_s_*(**r**,*t*) function, which provides the direct evidence of the Grotthuss mechanism. Over very short times (in femtosecond time scale), the proton moves at small distance within hydronium so that *G_s_*(**r**,*t*) shows only one maximum. This motion can mainly be attributed to "unproductive" proton transfers, including fast "proton rattling", i.e., recurrent proton-transfer events that occur within the non-transfer intervals [121]. At longer monitoring time (in picosecond time scale), the first maximum of *G_s_*(**r**,*t*) is gradually shifted to longer distances and then splits into two maxima (Figure 16). The position of the second maximum roughly corresponds to the size of the first hydration shell and changes little over time. However, the intensity of both maxima naturally decreases with time: in the long time limit, the system looses memory of the initial configuration and *G_s_*(**r**,*t*) becomes independent of the distance. It is natural to assume that the well-pronounced second maximum on the van Hove autocorrelation function is associated with the proton hopping along the network of hydrogen bonds, as schematically illustrated by the insert in Figure 16.

From the above discussion, it is reasonable to conclude that we obtained a direct confirmation that the charge transfer in the hydrophilic channel of the ionomeric membrane indeed proceeds via the Grotthuss mechanism. Of course, analysis of *G_s_*(**r**,*t*) provides information on the time and length scales of dynamic processes but not on different molecular structures that participate in the charge mobility process.

## Conclusion

Due to up-to-date demands on the polymer proton-exchange membranes with excellent mechanical characteristics, chemical stability, high proton conductivity, in-depth understanding the polymer microstructure and its connection with physical properties and fuel cell operating conditions is needed. Because the charge mobility can hardly become higher than in water or aqueous hydrochloric acid solution, the key challenge is to increase the charge mobility by optimizing the morphology of the membrane. Although a considerable experimental effort has been undertaken to study the morphology of hydrated Nafion, the microsegregated structure of this complex material is not well understood until now. This is primarily due to the ambiguous interpretation of experimental data. In this regard the role of computer modeling is particularly significant. Of course, it is impossible to solve this problem without using different mutually supplementary computational methods (quantum mechanical, atomistic, and mesoscopic) and the wide application of high-performance computing. This is a fundamentally multiscale materials science problem. In the present work, large-scale atomistic and first-principles molecular dynamics simulations were employed to investigate the structure formation in a hydrated Nafion membrane and the solvation and transport of protons in the water channel of the membrane. The most important findings from the research can be summarized as follows.

1. We have carried out atomistic force field-based simulations for water/Nafion systems, containing more than 4 million atoms, which are large enough to observe several hydrophilic domains. At water loading levels corresponding to the operating conditions, the predicted microphase-separated morphology can be classified as bicontinuous: both *majority* (hydrophobic) and *minority* (hydrophilic) subphases are three-dimensionally continuous and organized in an irregular ordered pattern, which is largely similar to that known for bicontinuous double-diamond structures. This behavior is due to the fact that Nafion is a typical amphiphilic polymer: it combines, in one macromolecule, strongly hydrophobic and strongly hydrophilic groups, which gives rise to a constrained hydrophobic/hydrophilic nanoseparation in the presence of physisorbed water. As a result, the developed morphology is generally close to the partly ordered mesophases or microemulsions characteristic of low-molecular-weight surfactant systems in a non-polar solvent. The existence of the well pronounced two-phase structural organization can explain in part the surprisingly high ionic conductivity of hydrated Nafion membranes even at relatively low water content. The characteristic size of the hydrophilic channels estimated from Bragg’s equation for water phase is about 25–50 Å, depending on water content. This is in reasonable agreement with the available experimental data for hydrated Nafion.

2. To clarify the issue of whether the driving force for ion dissociation (association) is dominantly of energetic or entropic nature, we have calculated the potential of mean force (PMF), which characterizes the interaction between hydronium cations and oppositely charged sulfonate groups of Nafion, and examined its temperature dependence. The energetic and entropic contributions were found using a thermodynamic decomposition of PMF. It was shown that ion association observed with decreasing temperature is largely an entropic effect related to the loss of low-frequency modes. Because these modes are mainly responsible for transport processes, one can expect that the entropic effects would play an important role in ionic conductivity. Although at temperatures substantially below room temperature, significant ion association does exist, the cations are still mobile and contribute to ionic conductivity.

3. For a relatively small system consisting of sulfonated Nafion monomers and water molecules, it was shown that quantum molecular dynamics simulations are able to predict the microsegregation of water and Nafion: water molecules, hydrated proton complexes, and sulfonic groups are arranged in the hydrophilic regions surrounded by the hydrophobic sections of Nafion and thus the two-phase structure is maintained.

4. Based on the results from our atomistic simulation, a realistic model of an ion-conducting channel containing Nafion chains and water molecules was designed and studied using quantum molecular dynamics. Proton solvation is only fairly understood in bulk water, but has not been comprehended in the ion-conducting channels of proton conducting ionomer membrane. This contrasts with the importance of this process for membrane transport. We have found that the proton exchange between hydronium and water molecules is a relatively fast process as compared to the formation of both hydronium and Eigen ions. The formation processes of hydronium and Eigen ions are strongly correlated, while the mutual transformations of hydronioum/Zundel ions and Zundel/Eigen ions are less correlated processes. From the results of the QMD simulations, one can expect that for our model of the ion-conducting nanochannel, proton transfer through Zundel–Eigen–Zundel transformations should dominate.

5. Our extensive 120 ps-long density functional theory (DFT)-based simulations of charge migration in the 1200-atom model of the hydrophilic Nafion nanochannel allowed us to observe the bimodality of the van Hove autocorrelation function *G_s_*(**r**,*t*), which provides the direct evidence of the Grotthuss bond-exchange (hopping) mechanism as a significant contributor to the proton conductivity. Over very short times (in femtosecond time scale), the proton moves at small distance within the hydronium so that *G_s_*(**r**,*t*) shows only one maximum. At longer monitoring times (in picosecond time scale), this maximum gradually shifts to longer distances and then splits into two maxima, the second of which indicates the proton hopping along the network of hydrogen bonds.
